# Supplementary material for: Wastewater plumes can act as non-physical barriers for migrating silver eel
Source: PLoS One. 2023 Jun 27;18(6):e0287189. doi: 10.1371/journal.pone.0287189 (PMC10298781; doi:10.1371/journal.pone.0287189)
Supplement: S1 Appendix — (PDF) [file pone.0287189.s001.pdf]

Wastewater plumes can act as non-physical barriers for migrating eel.

A field study based on telemetry and plume modelling

H.V. Winter[1]*, O.A. van Keeken[1], F. Kleissen[2], E.M. Foekema[1,3]

[1]Wageningen Marine Research, IJmuiden, the Netherlands

[2]Deltares, Delft, the Netherlands

[3] Wageningen University, Marine Animal Ecology group, Wageningen, The Netherlands

*Corresponding author: erwin.winter@wur.nl

Supporting information
Eel movements 2009

Characterisation of behaviour 2009 silver eel

Eel 2009	Behavioural code	Short description
1	2B	Diversion at plume
2	2B	Diversion at plume
3	?	Unclear
4	2A	Turning, no passage
5	1B	No response, passing through
6	1B	Turning, no passage
7	2B	Turning, eventually passage
8	2C	Turning, eventually passage
9	1A	No response, passing through
10	1A	No response, passing through
11	2B	Diversion at plume
12	1A	No response, passing through
13	-	Not detected
14	1A	No response, passing through
15	1A	No response, passing through
16	-	Not detected
17	2C	Diversion at plume
18	2B	Turning, eventually passage
19	2A	Turning, no passage
20	2B	Turning, eventually passage

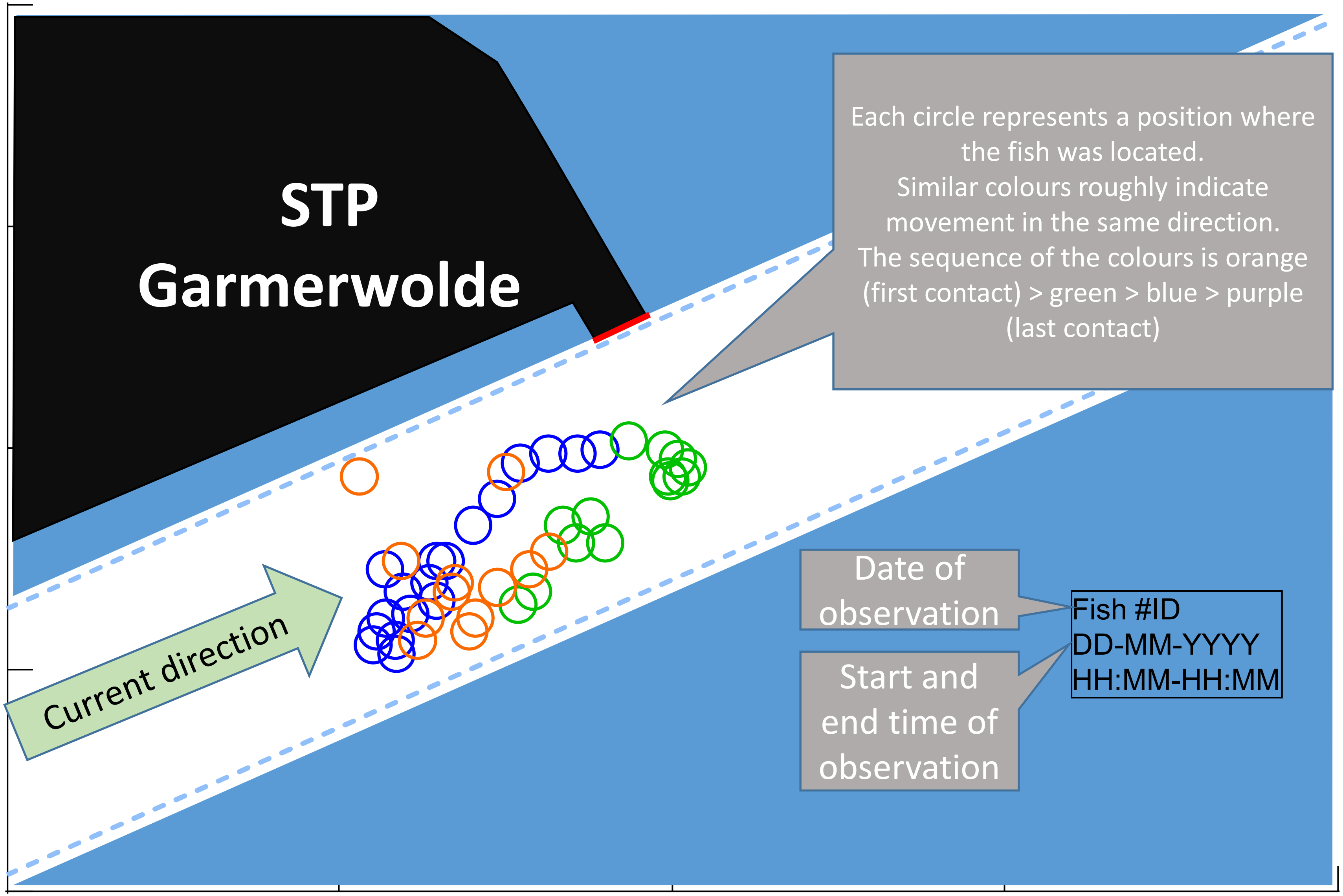
STP
Garmerwolde
Each circle represents a position where the fish was located.
Similar colours roughly indicate movement in the same direction.
The sequence of the colours is orange (first contact) > green > blue > purple (last contact)
Current direction
Date of observation
Start and end time of observation
Fish #ID
DD-MM-YYYY
HH:MM-HH:MM

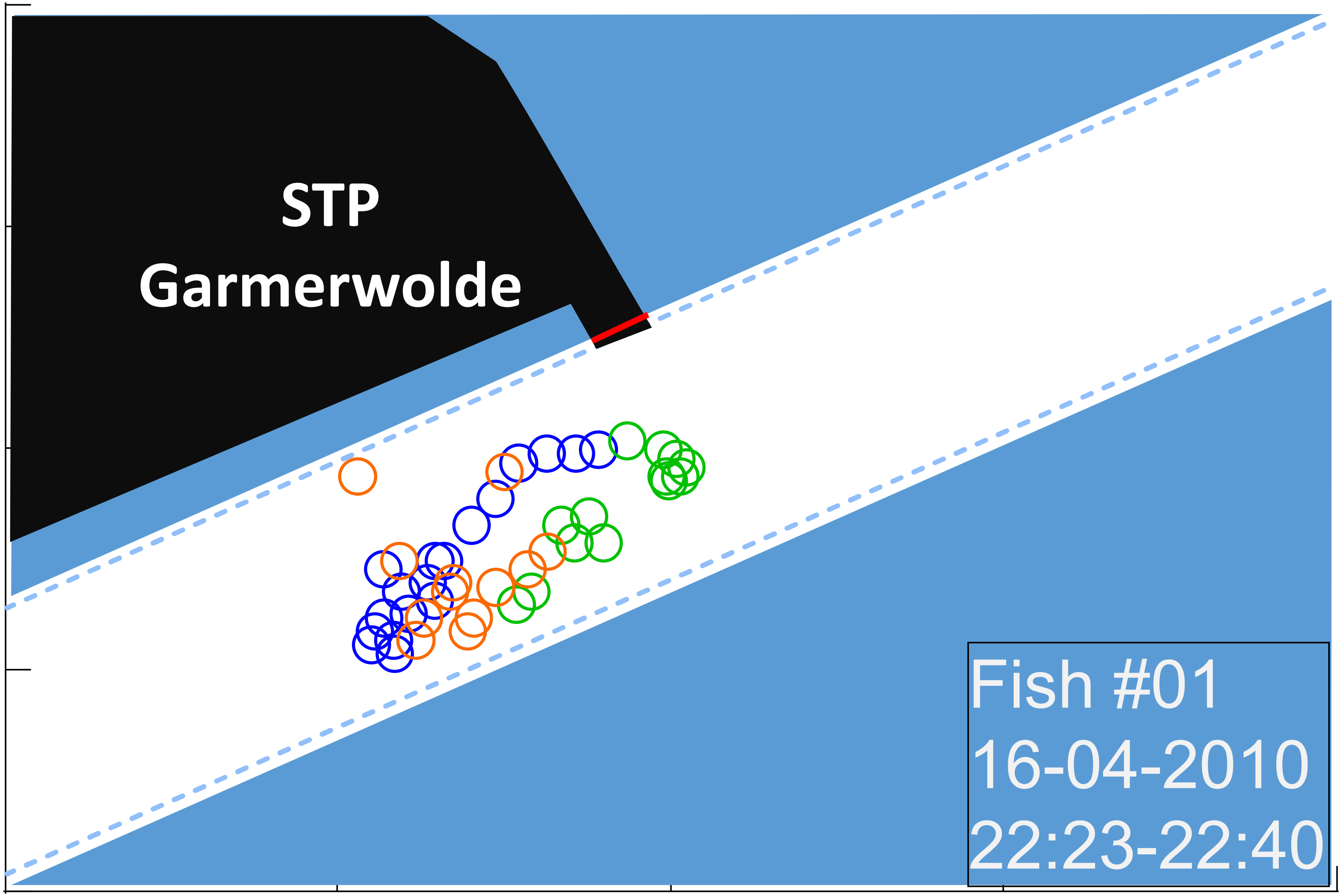

STP
Garmerwolde
Fish #01
16-04-2010
22:23-22:40

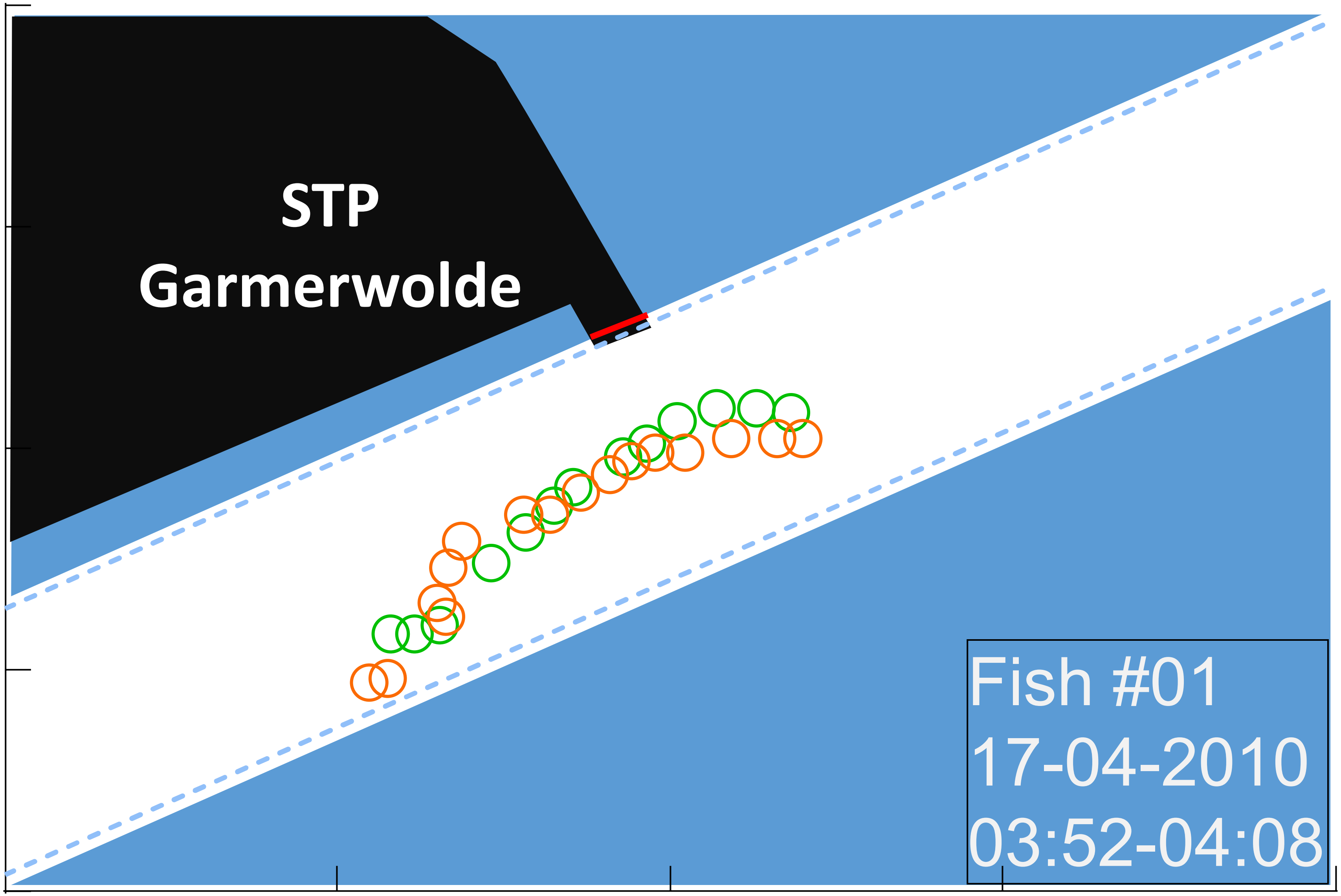

STP
Garmerwolde
Fish #01
17-04-2010
03:52-04:08

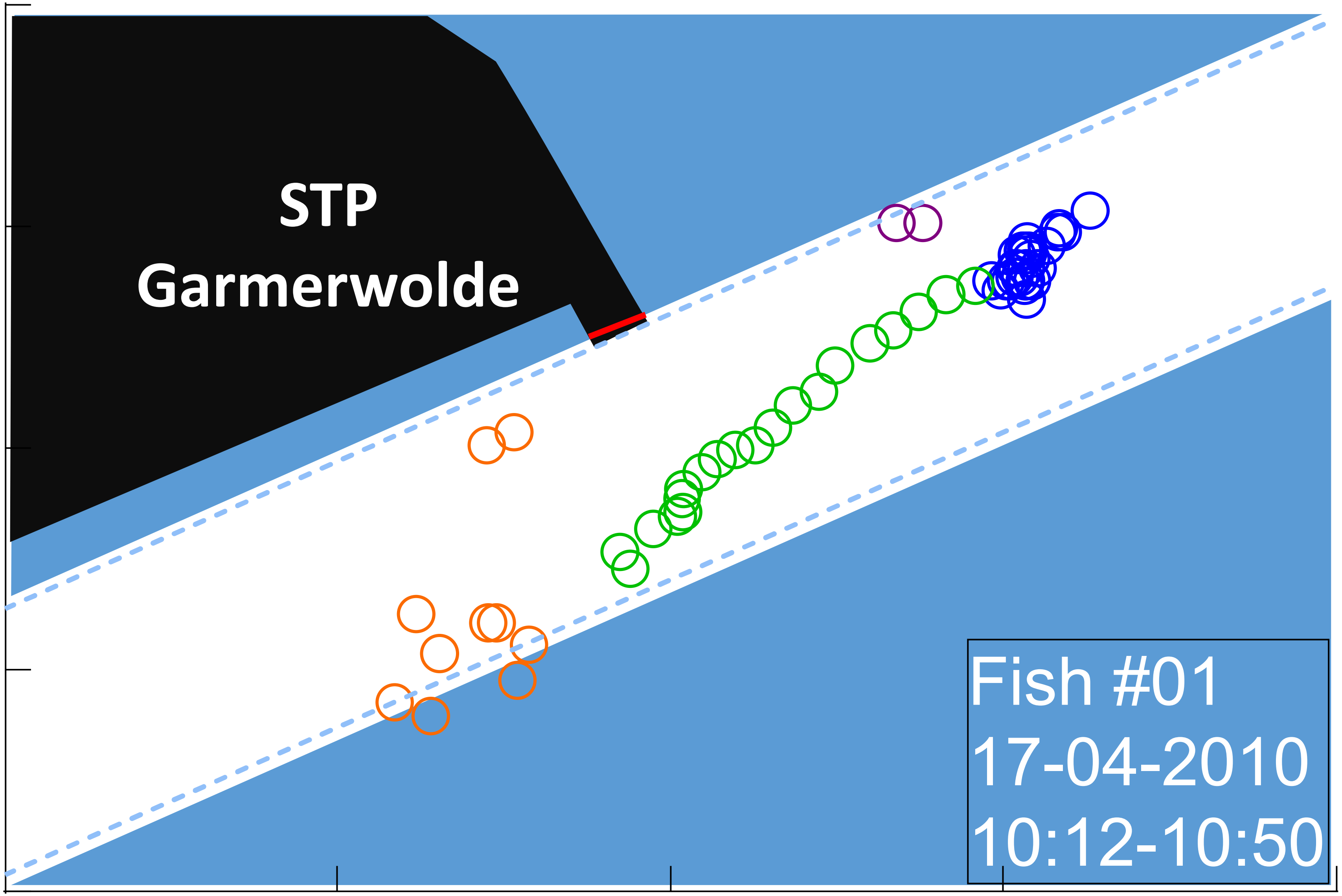
STP
Garmerwolde
Fish #01
17-04-2010
10:12-10:50

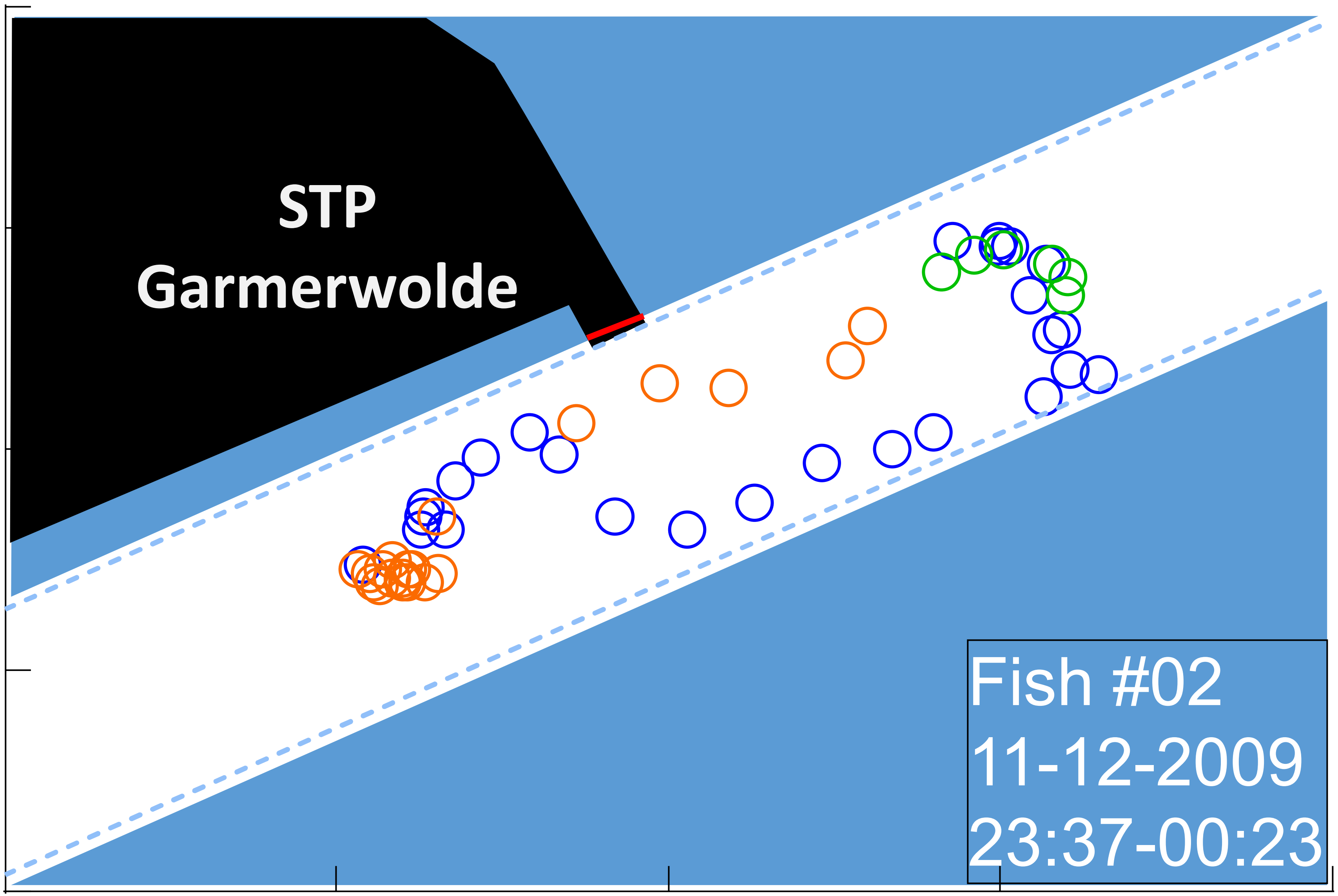
STP
Garmerwolde
Fish #02
11-12-2009
23:37-00:23

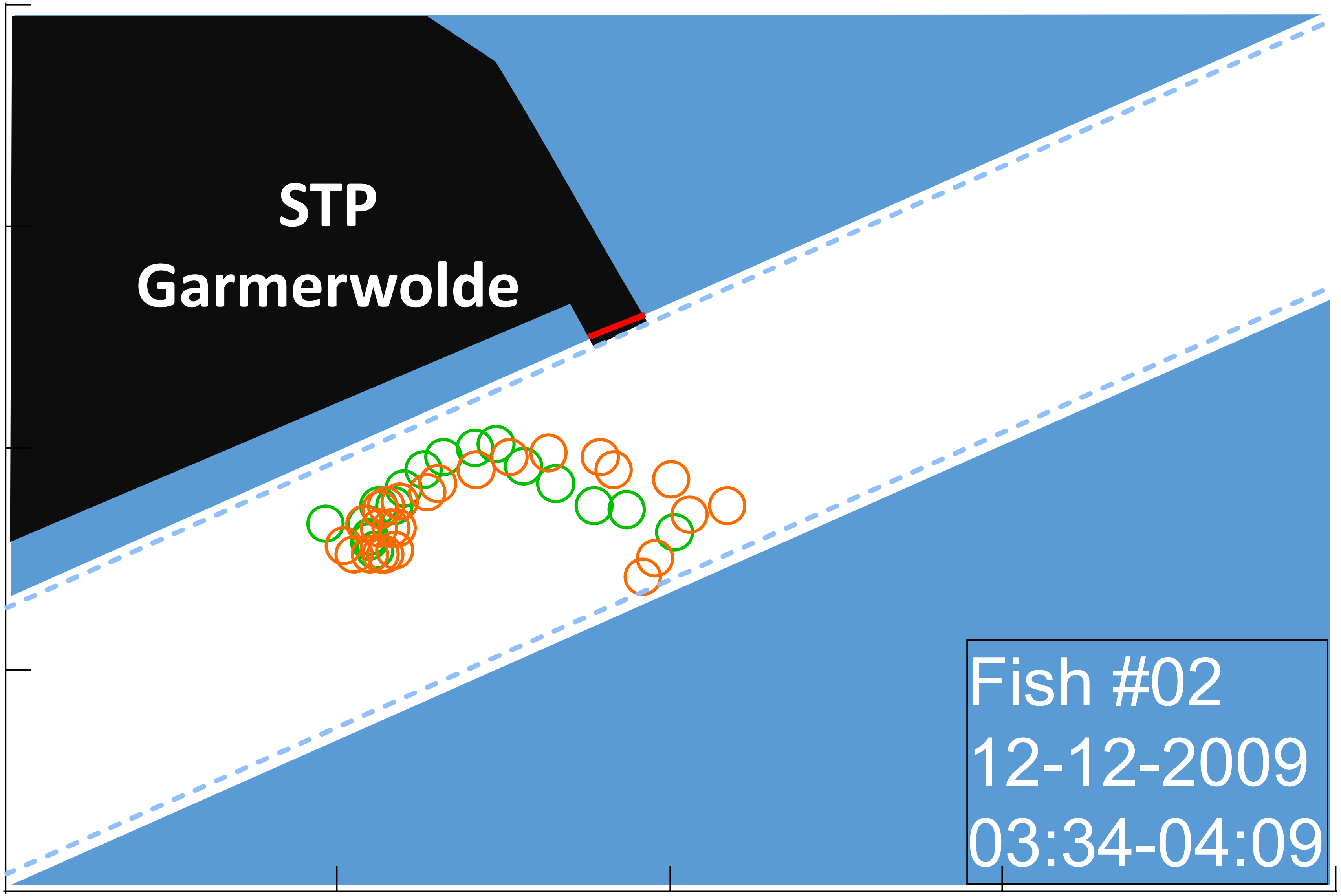

STP
Garmerwolde
Fish #02
12-12-2009
03:34-04:09

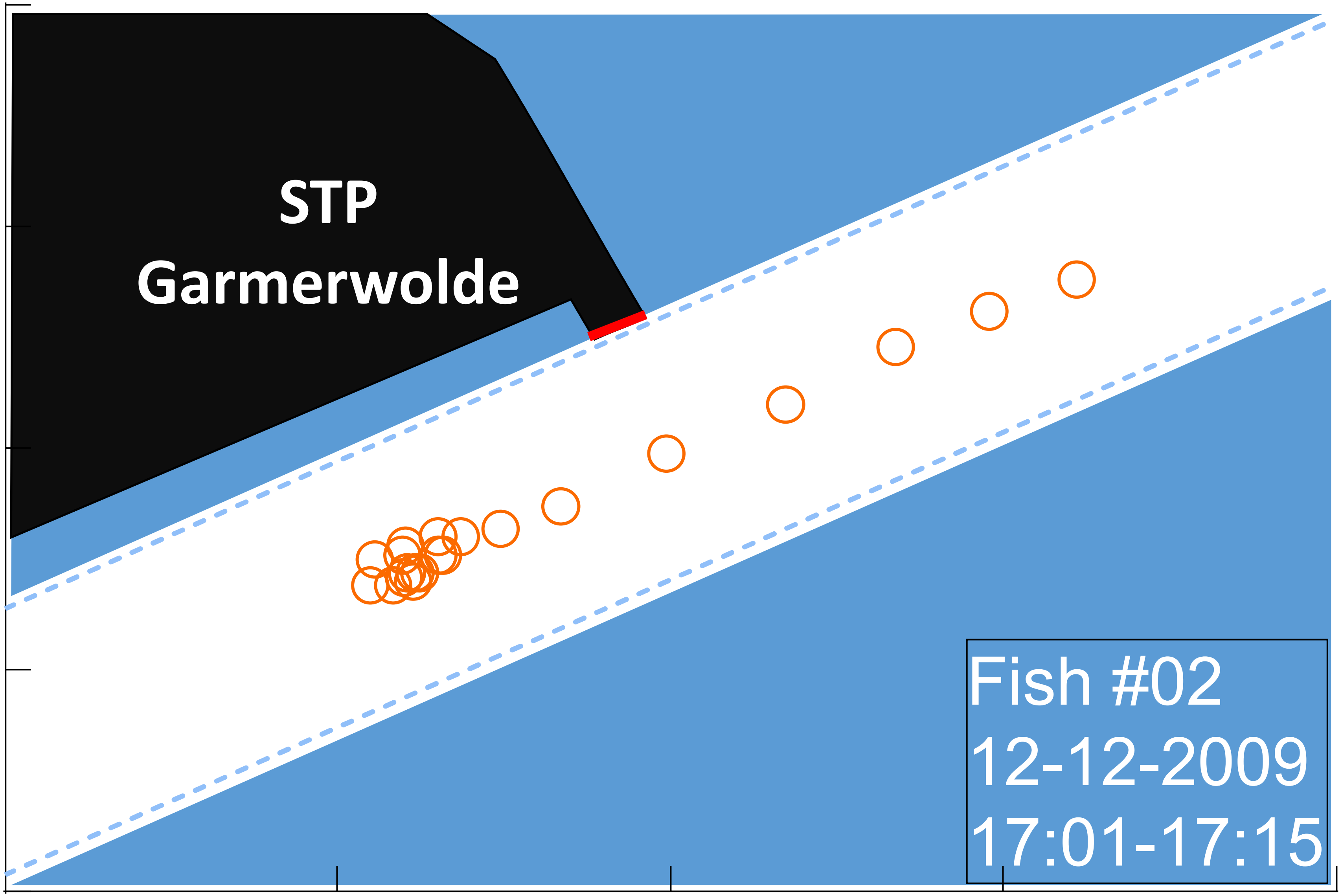
STP
Garmerwolde
Fish #02
12-12-2009
17:01-17:15

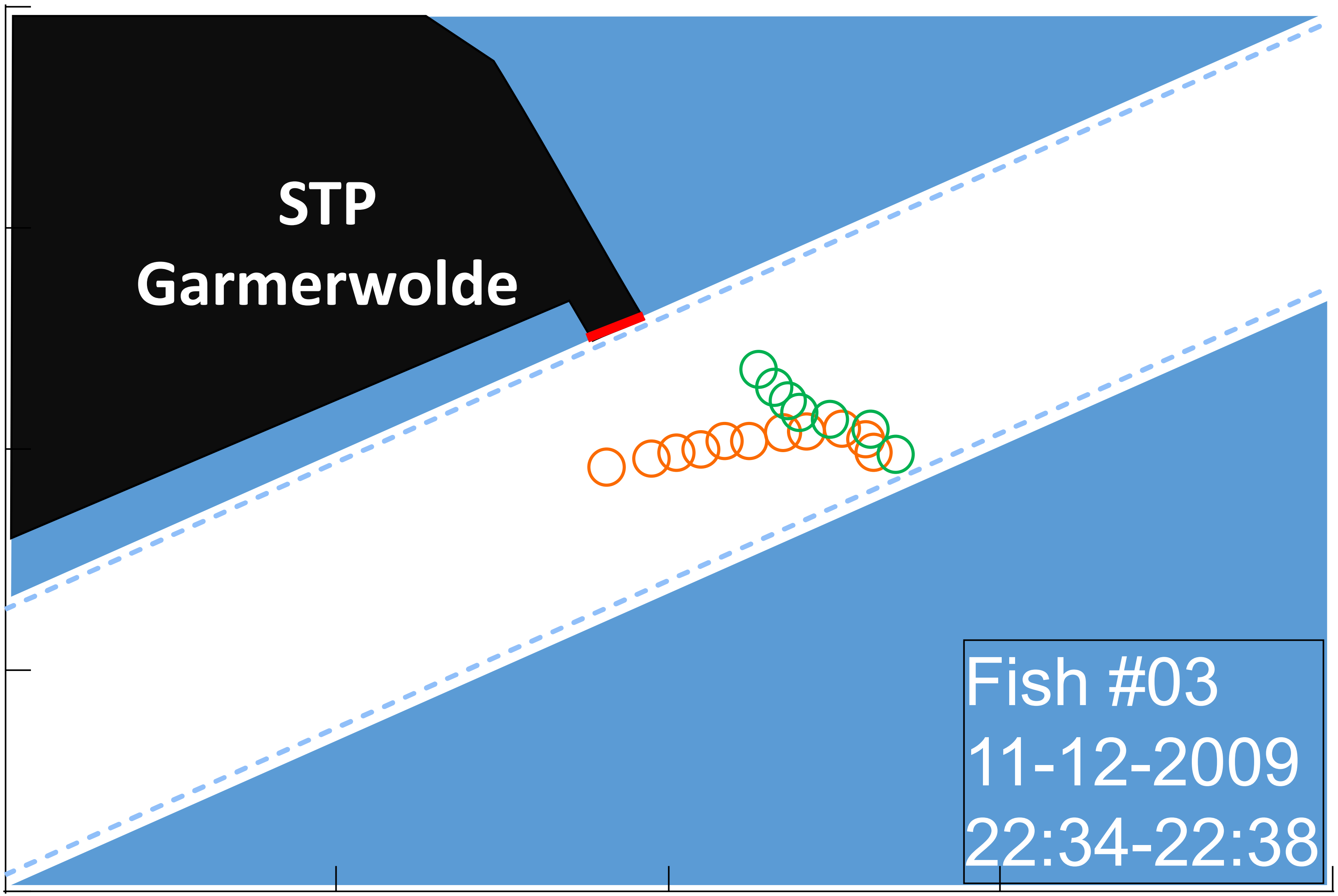

STP
Garmerwolde
Fish #03
11-12-2009
22:34-22:38

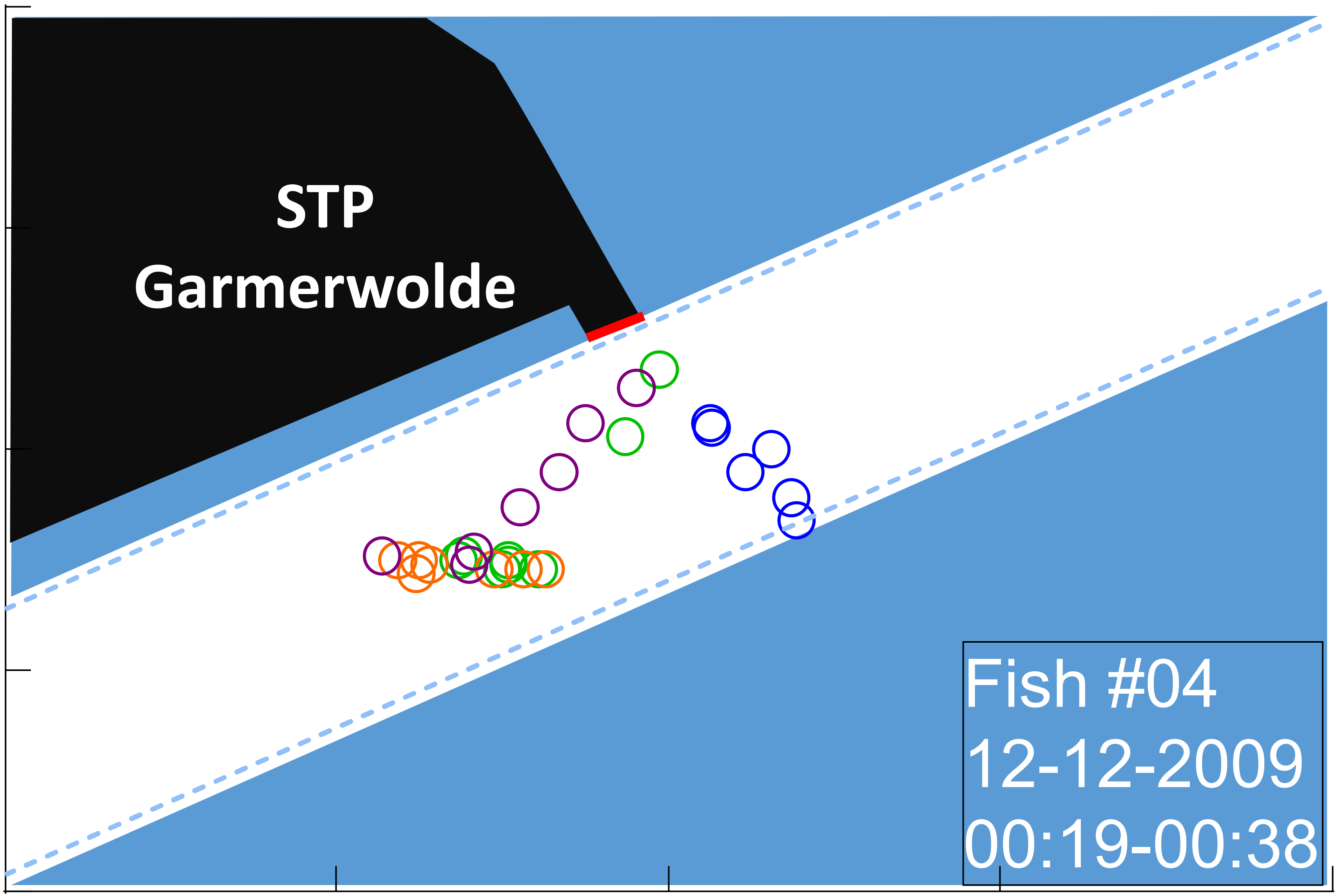
STP
Garmerwolde
Fish #04
12-12-2009
00:19-00:38

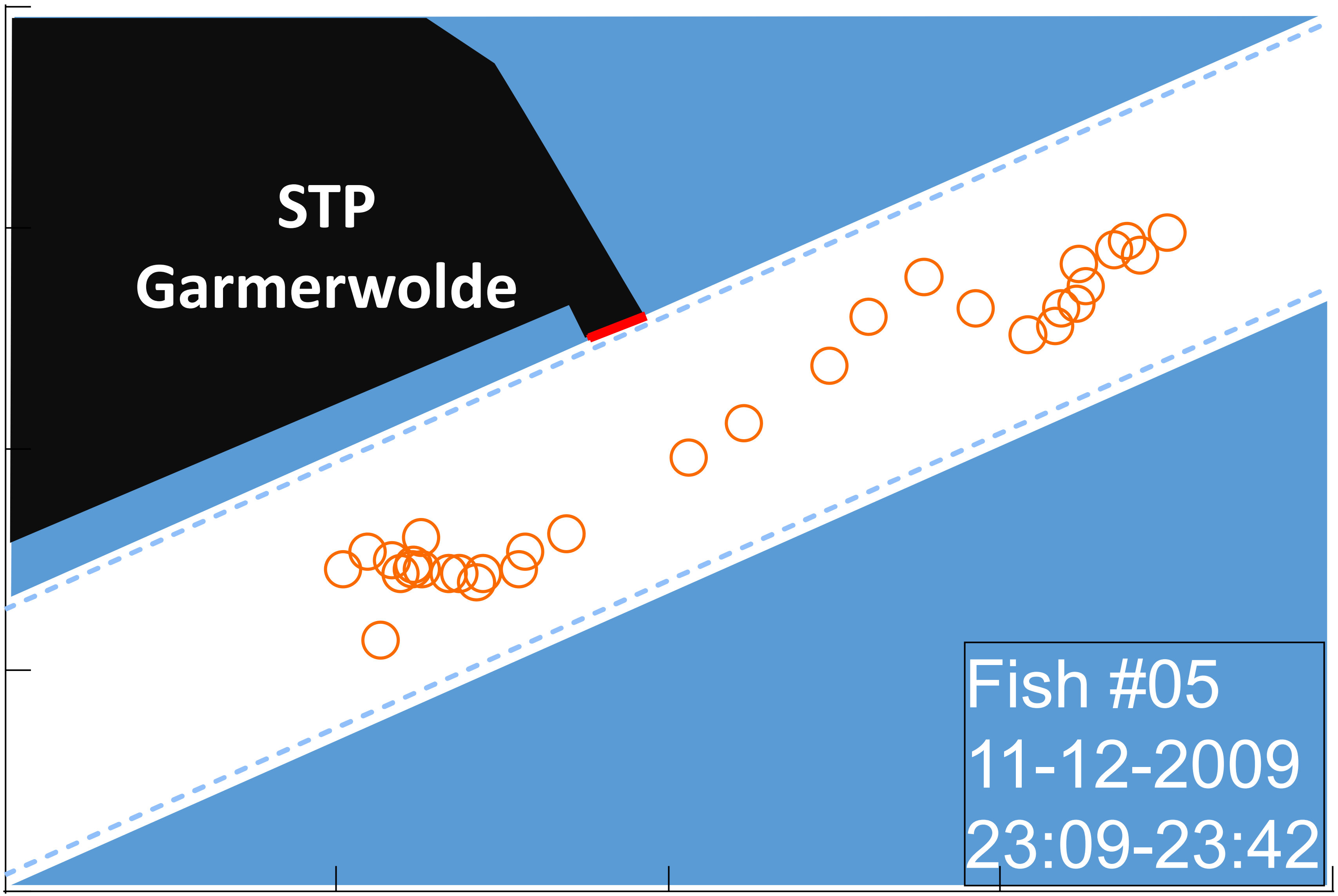
STP
Garmerwolde
Fish #05
11-12-2009
23:09-23:42

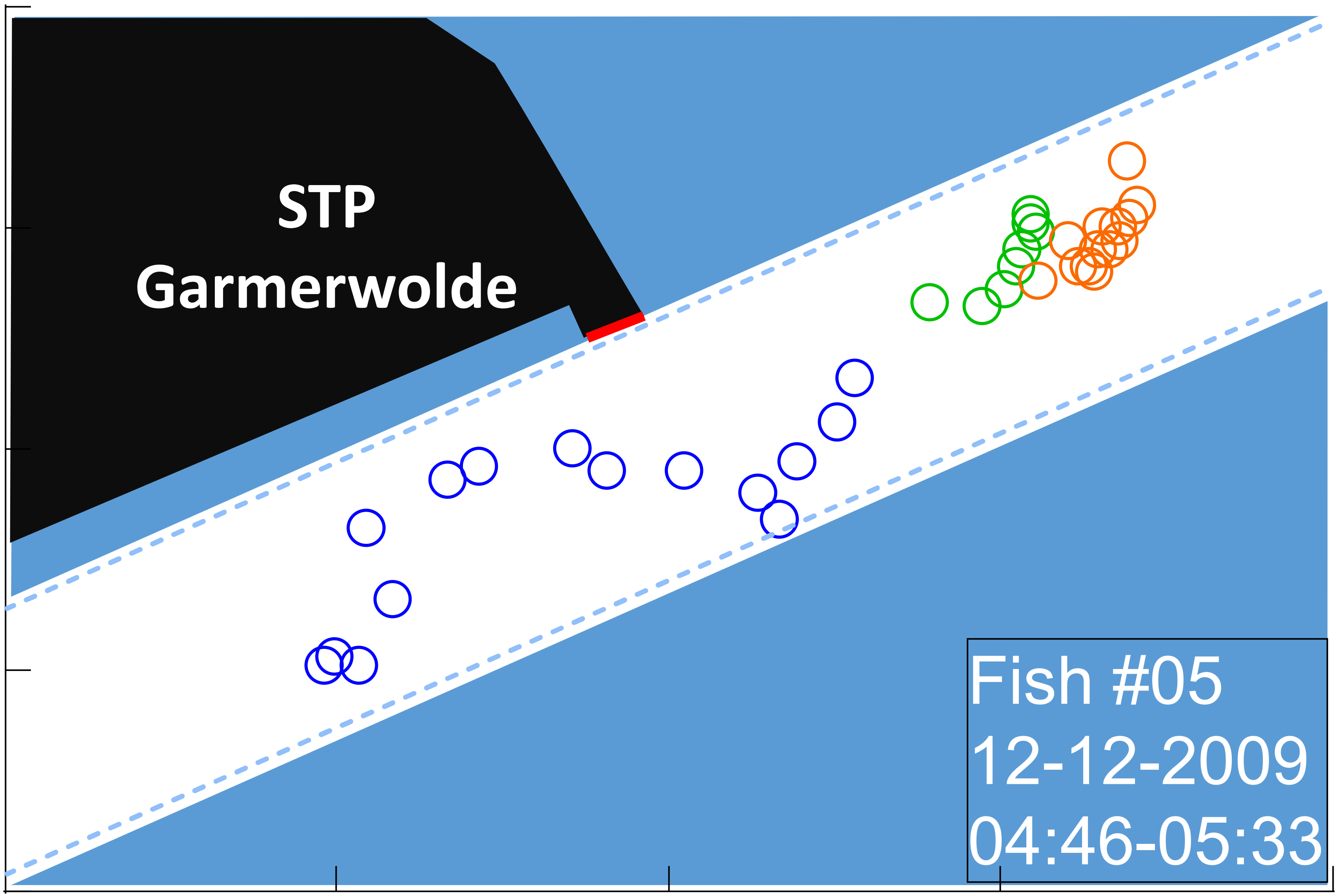

STP
Garmerwolde
Fish #05
12-12-2009
04:46-05:33

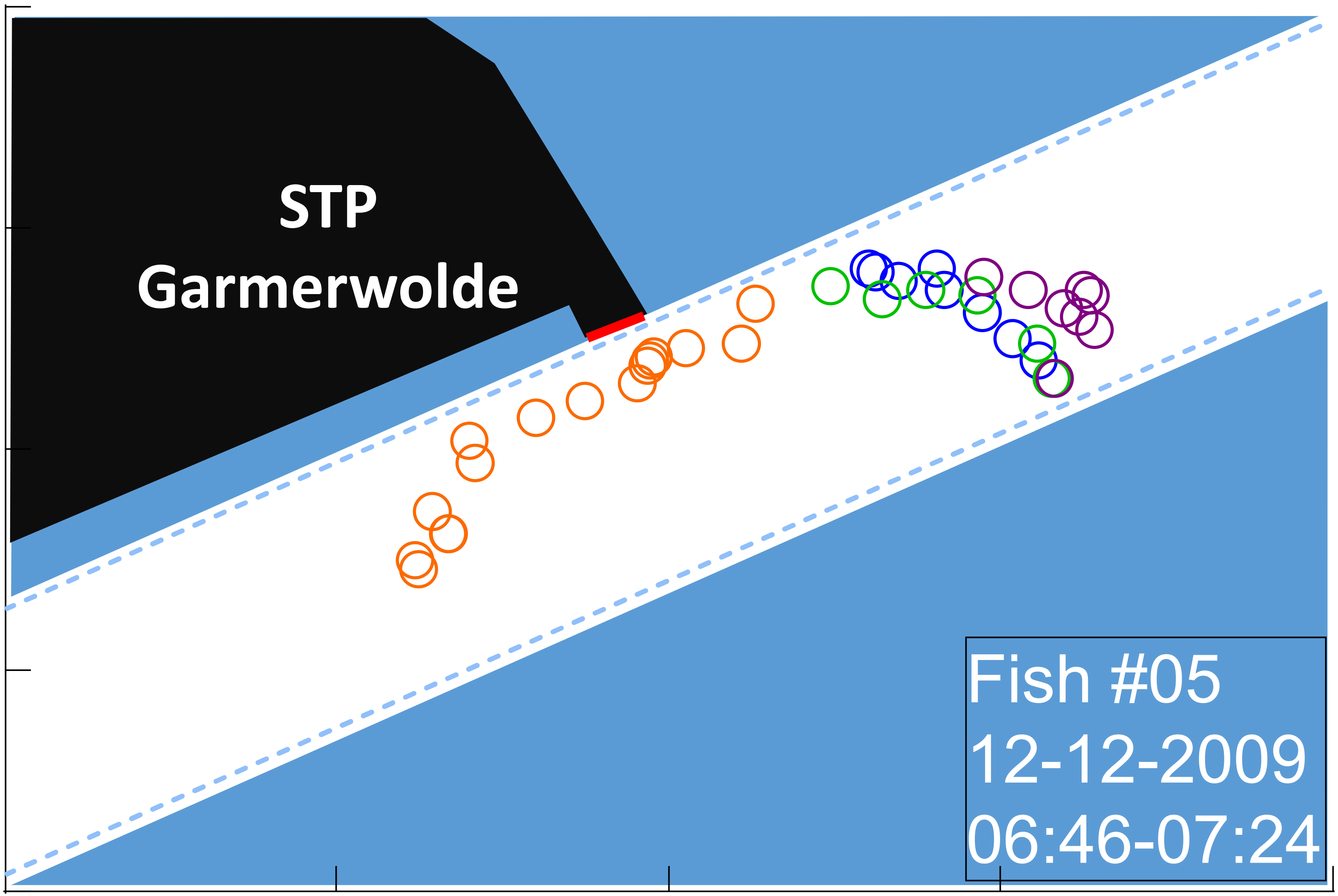
STP
Garmerwolde
Fish #05
12-12-2009
06:46-07:24

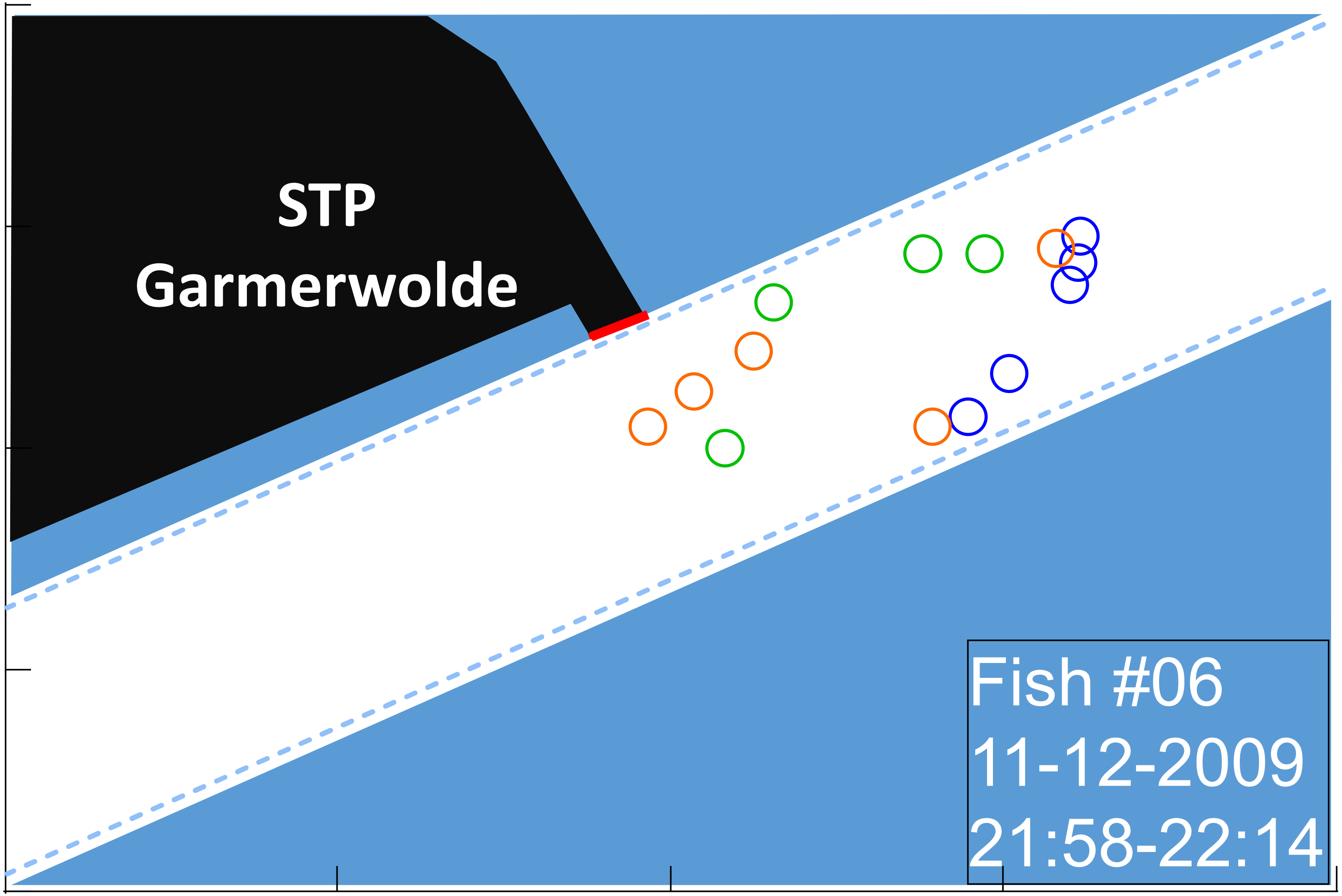
STP
Garmerwolde
Fish #06
11-12-2009
21:58-22:14

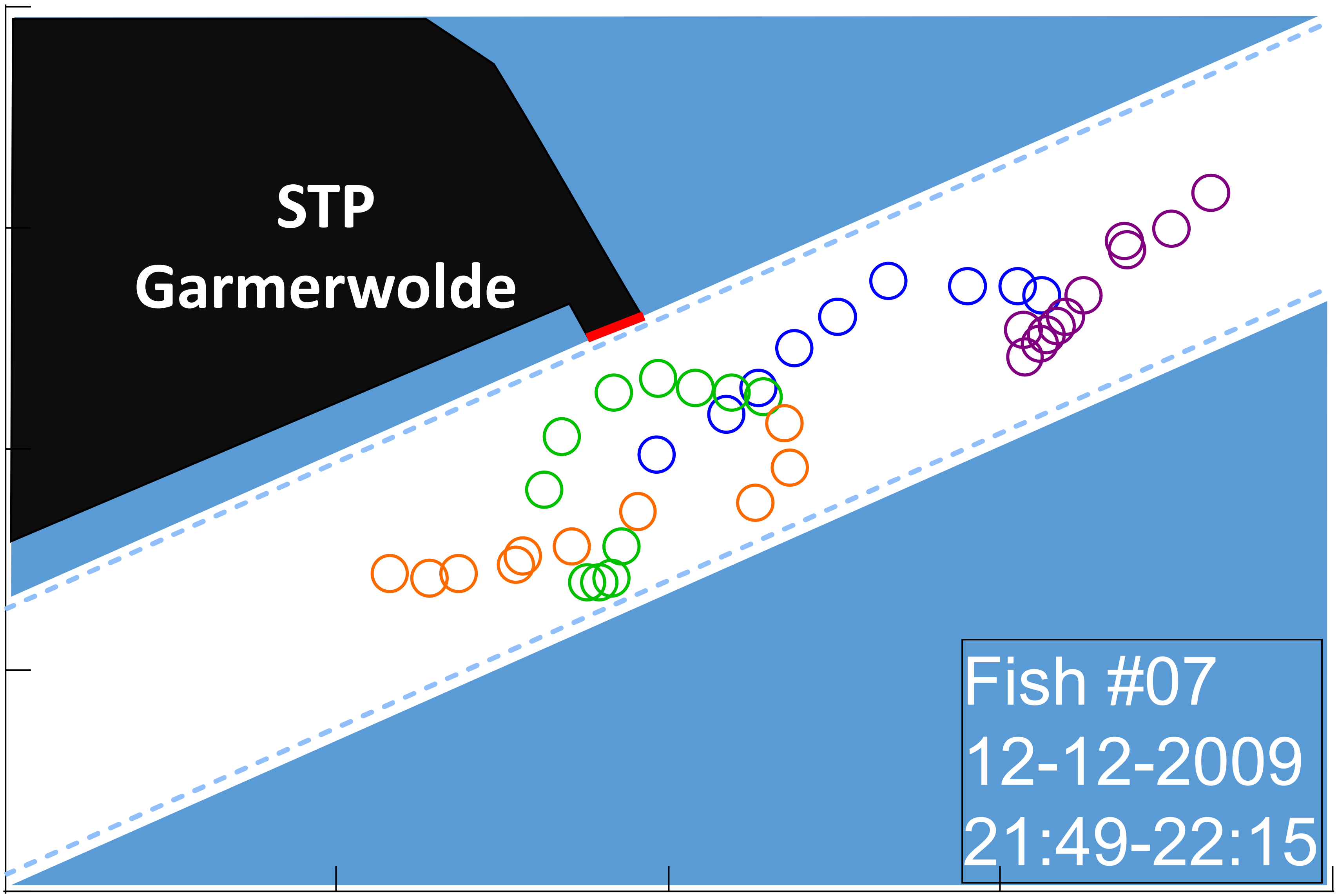
STP
Garmerwolde
Fish #07
12-12-2009
21:49-22:15

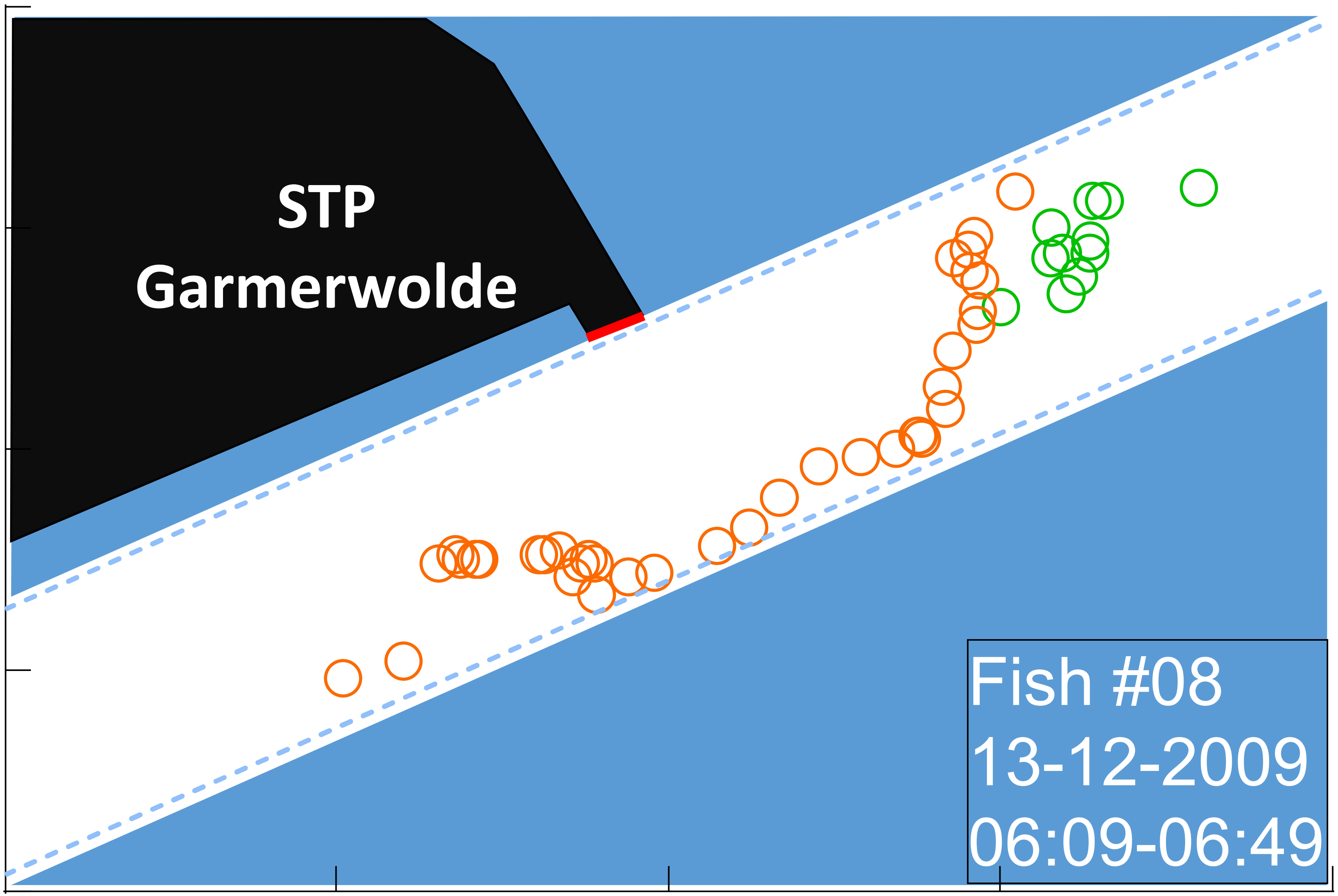

STP
Garmerwolde
Fish #08
13-12-2009
06:09-06:49

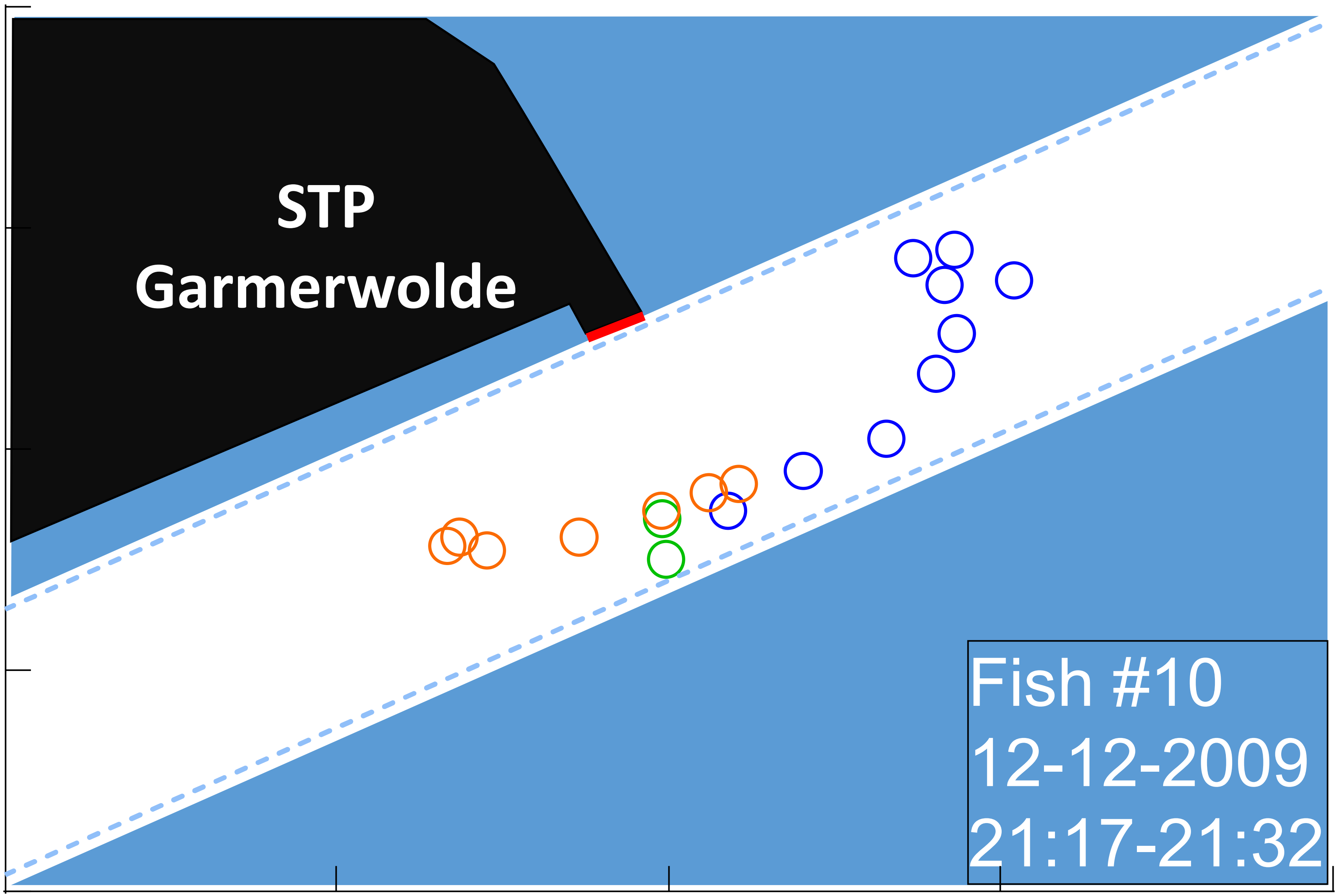
STP
Garmerwolde
Fish #10
12-12-2009
21:17-21:32

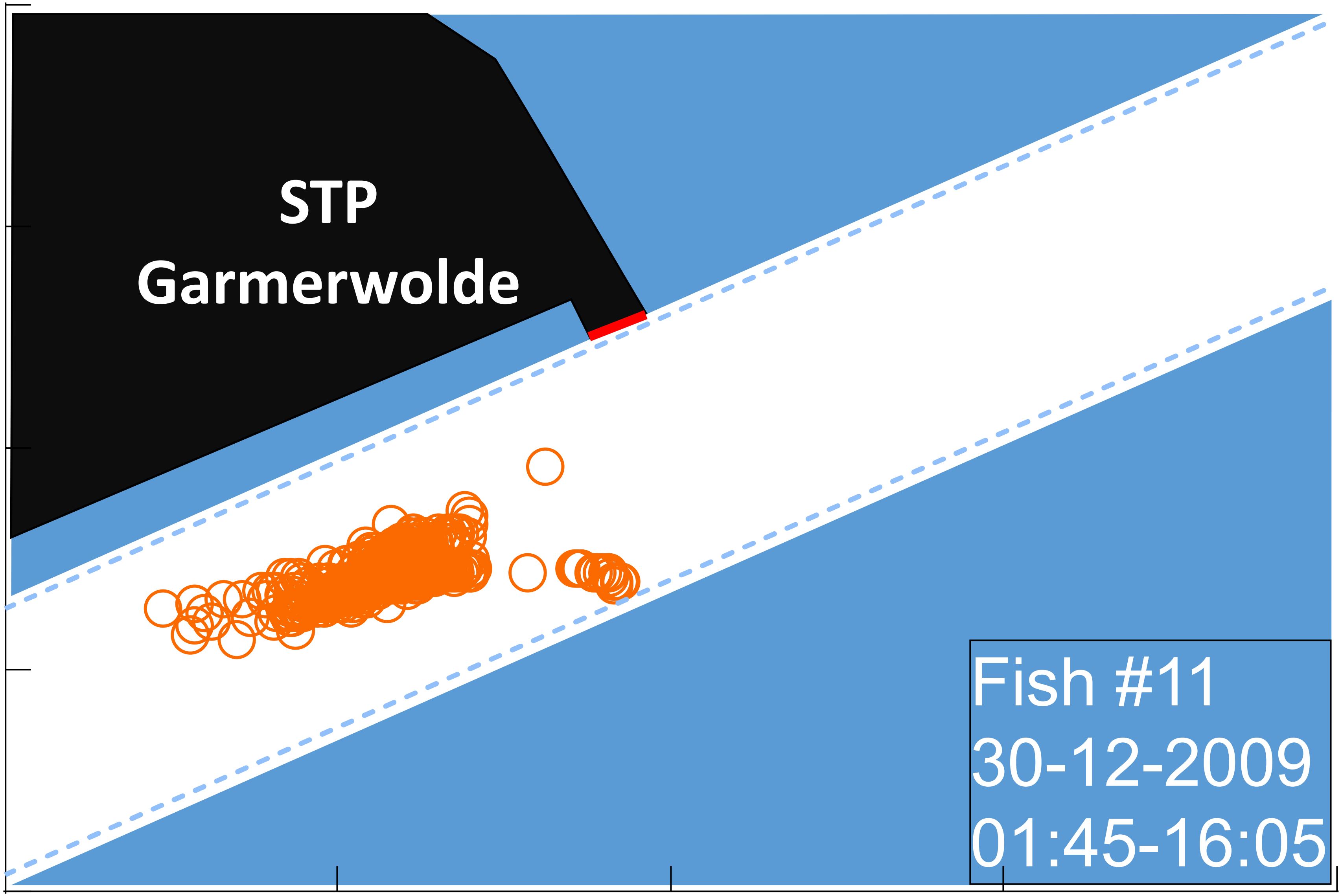
STP
Garmerwolde
Fish #11
30-12-2009
01:45-16:05

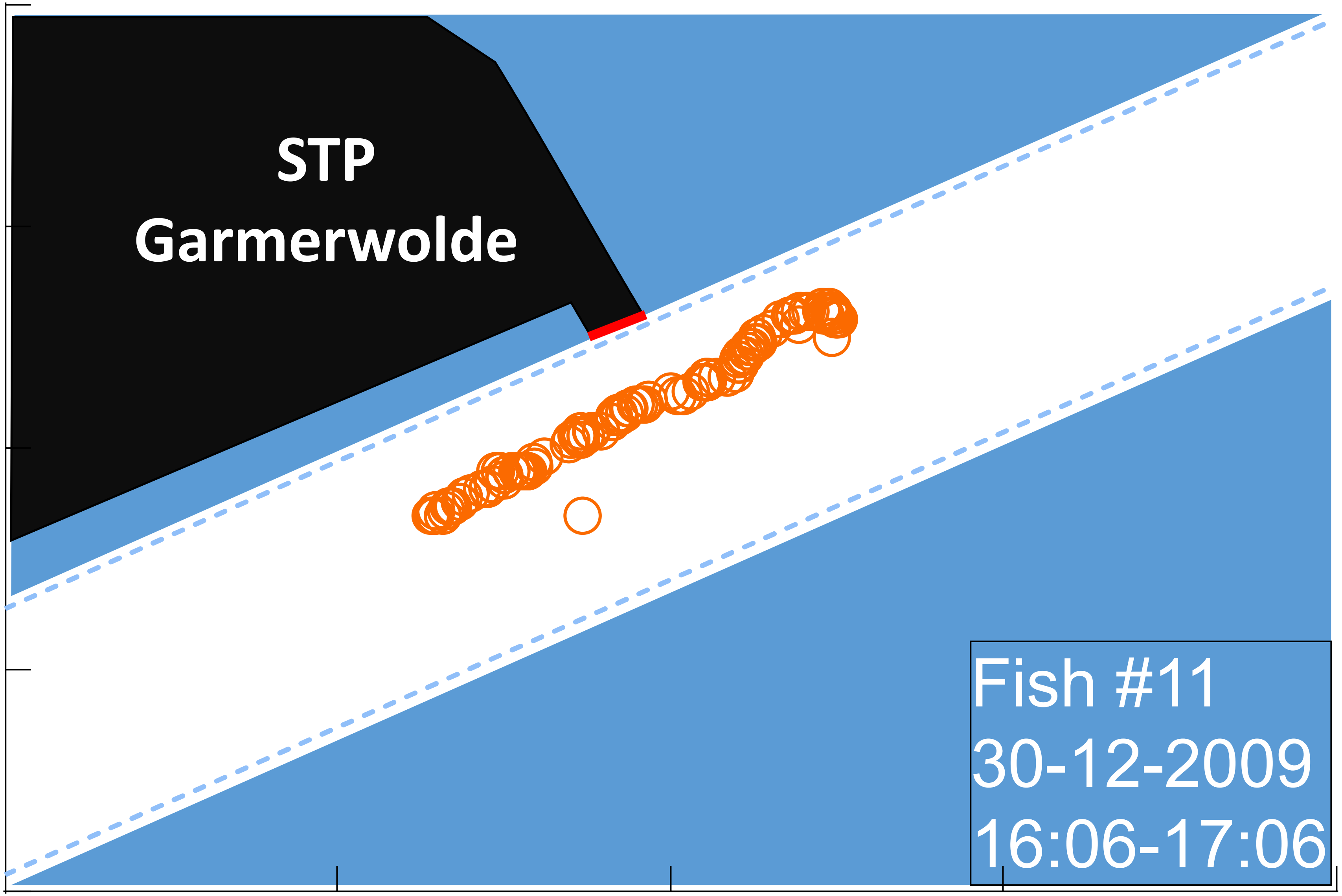
STP
Garmerwolde
Fish #11
30-12-2009
16:06-17:06

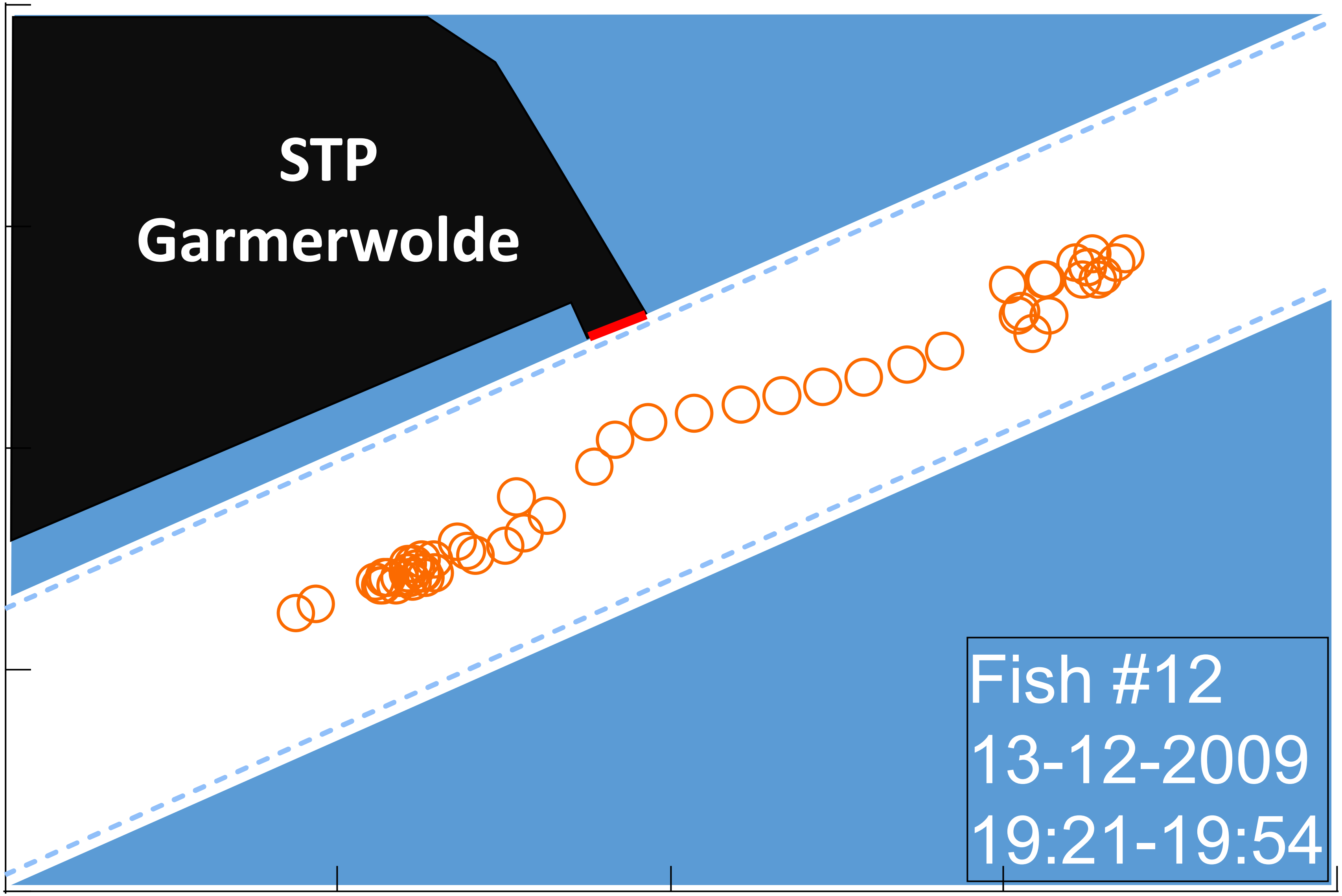
STP
Garmerwolde
Fish #12
13-12-2009
19:21-19:54

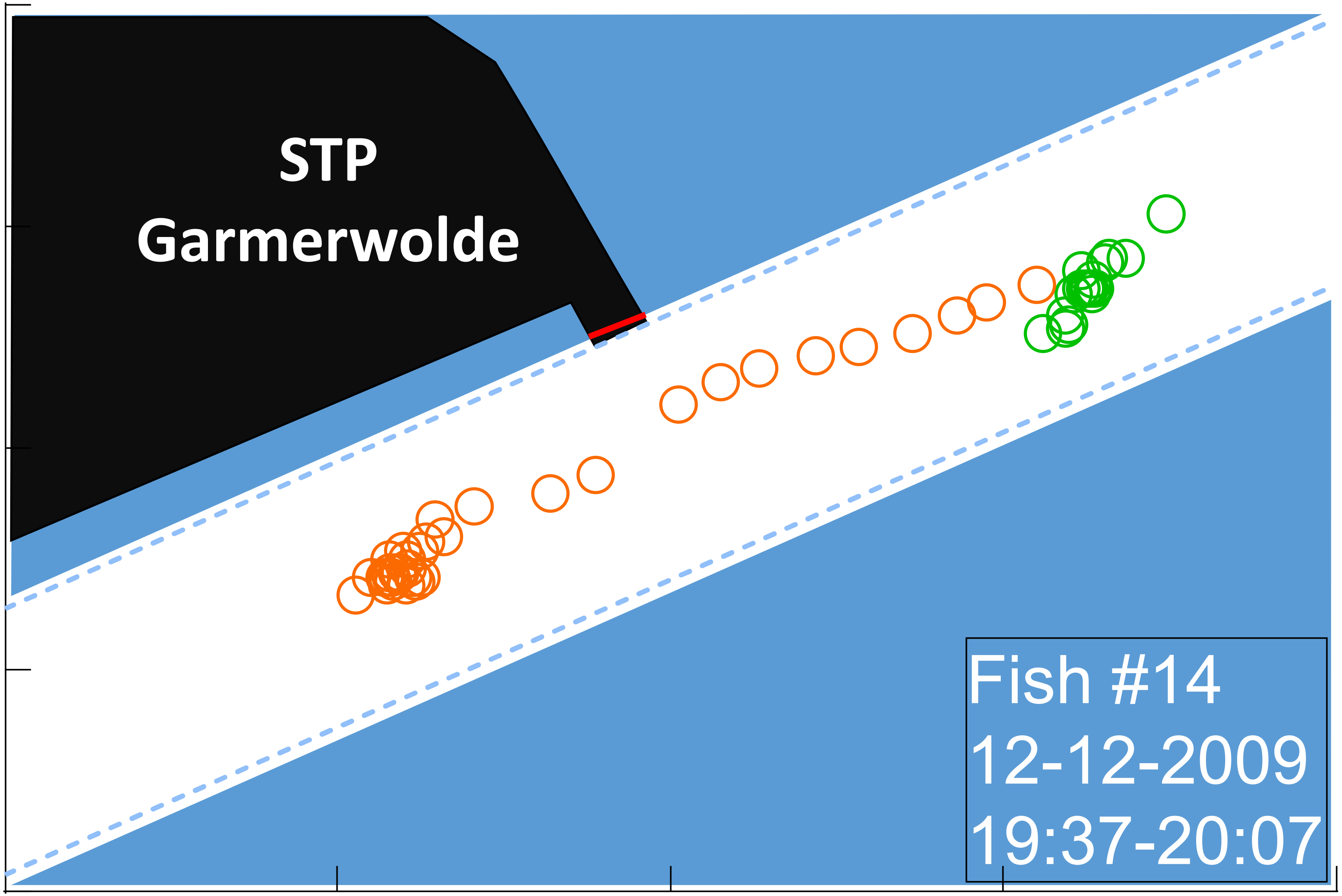
STP
Garmerwolde
Fish #14
12-12-2009
19:37-20:07

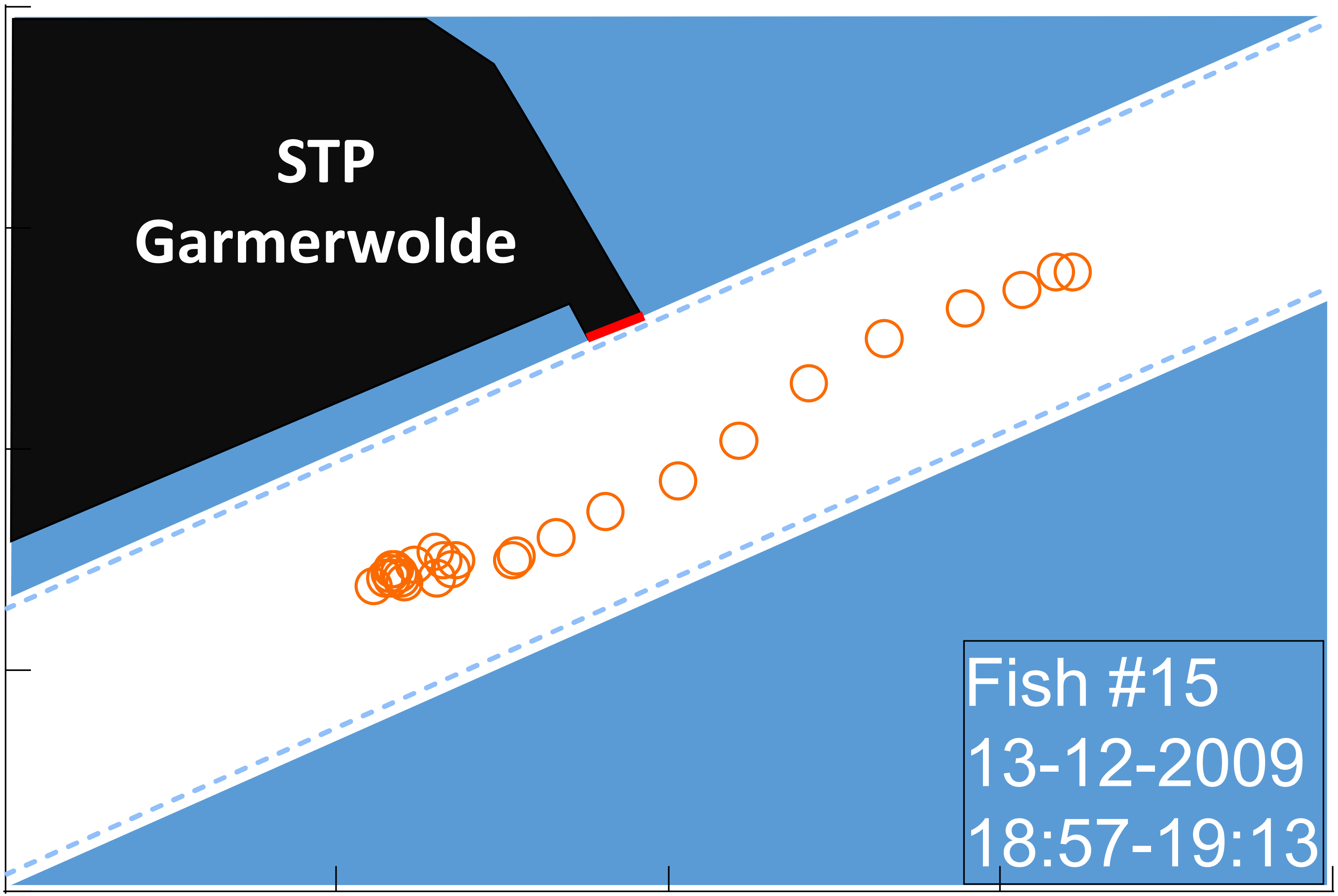
STP
Garmerwolde
Fish #15
13-12-2009
18:57-19:13

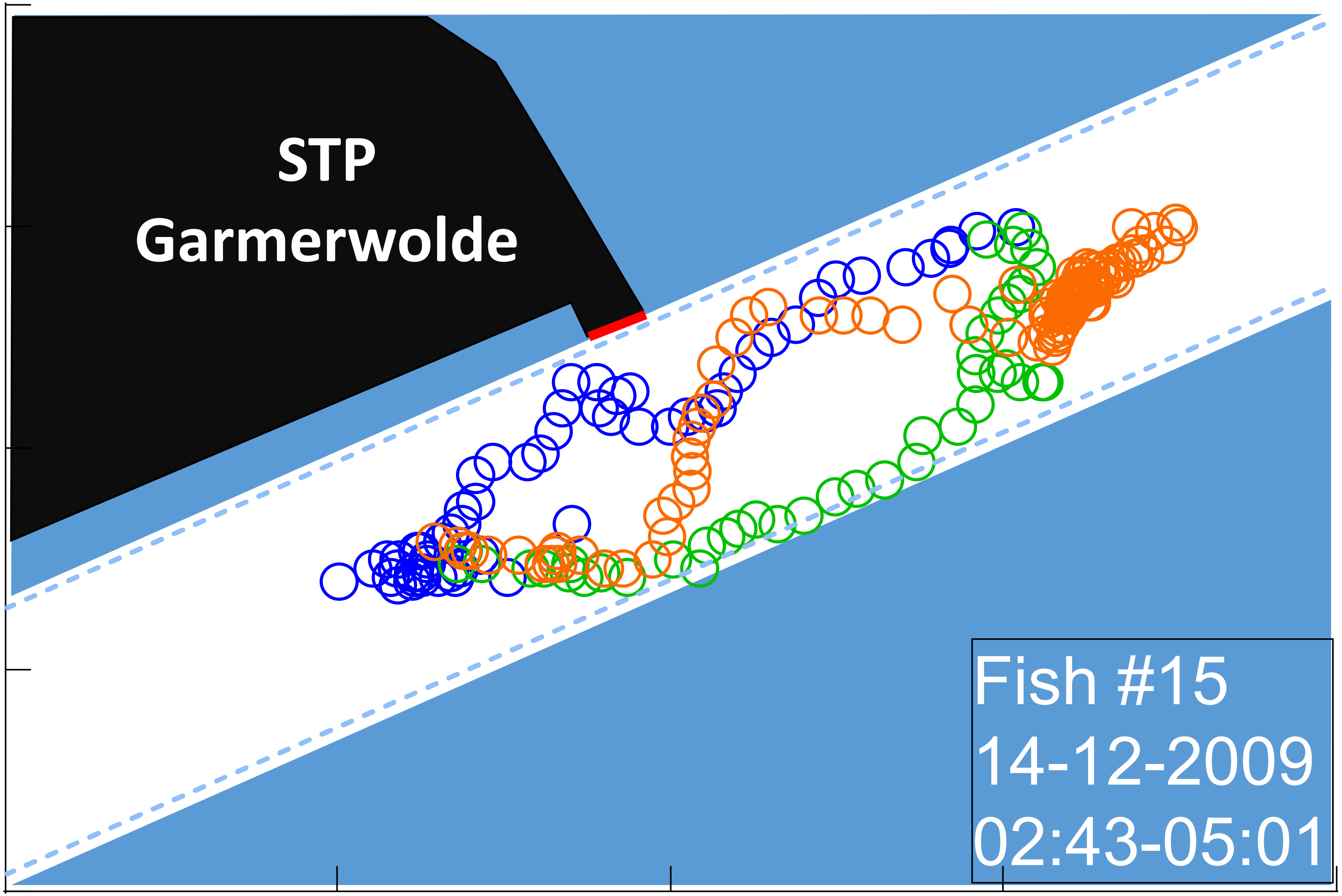
STP
Garmerwolde
Fish #15
14-12-2009
02:43-05:01

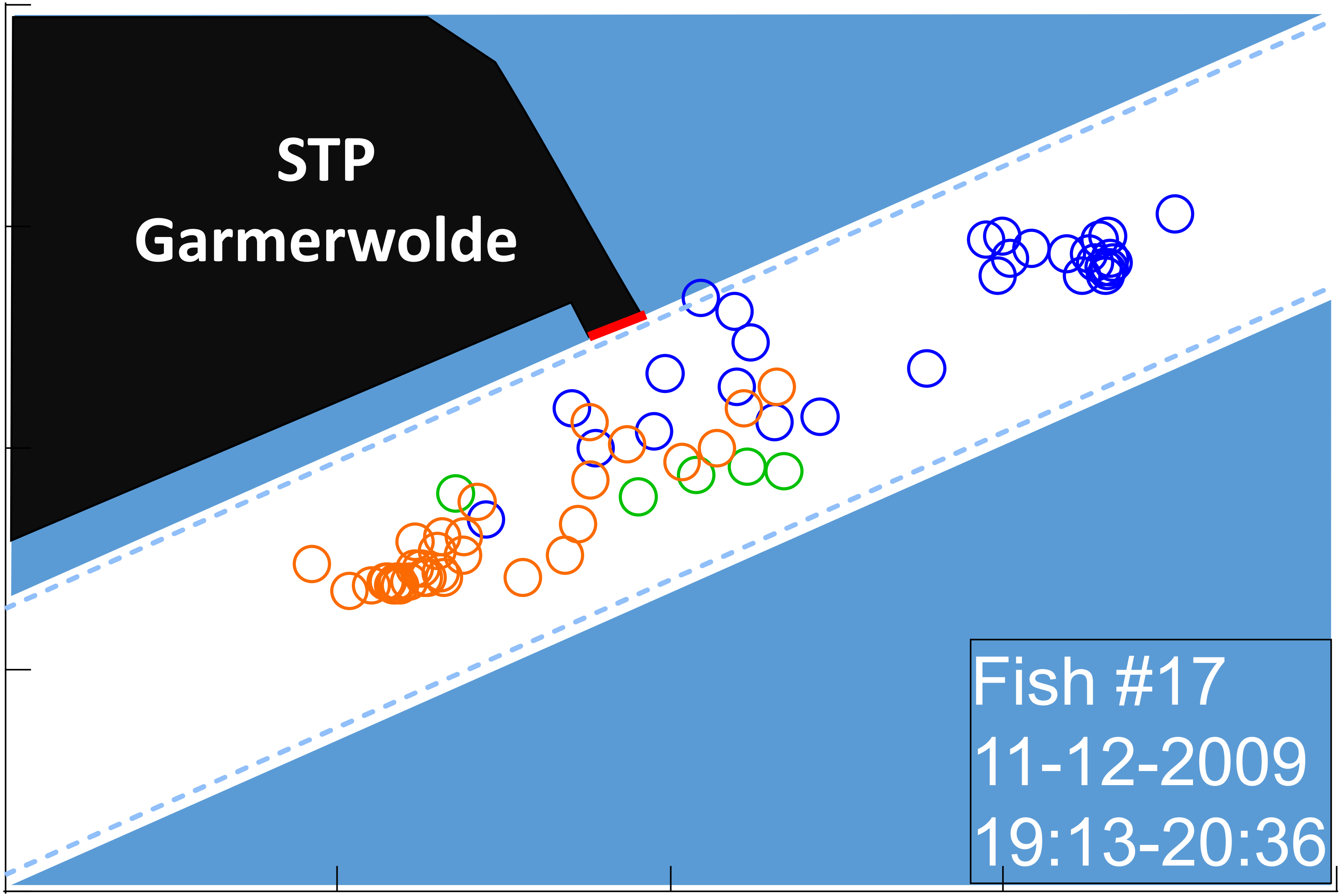
STP
Garmerwolde
Fish #17
11-12-2009
19:13-20:36

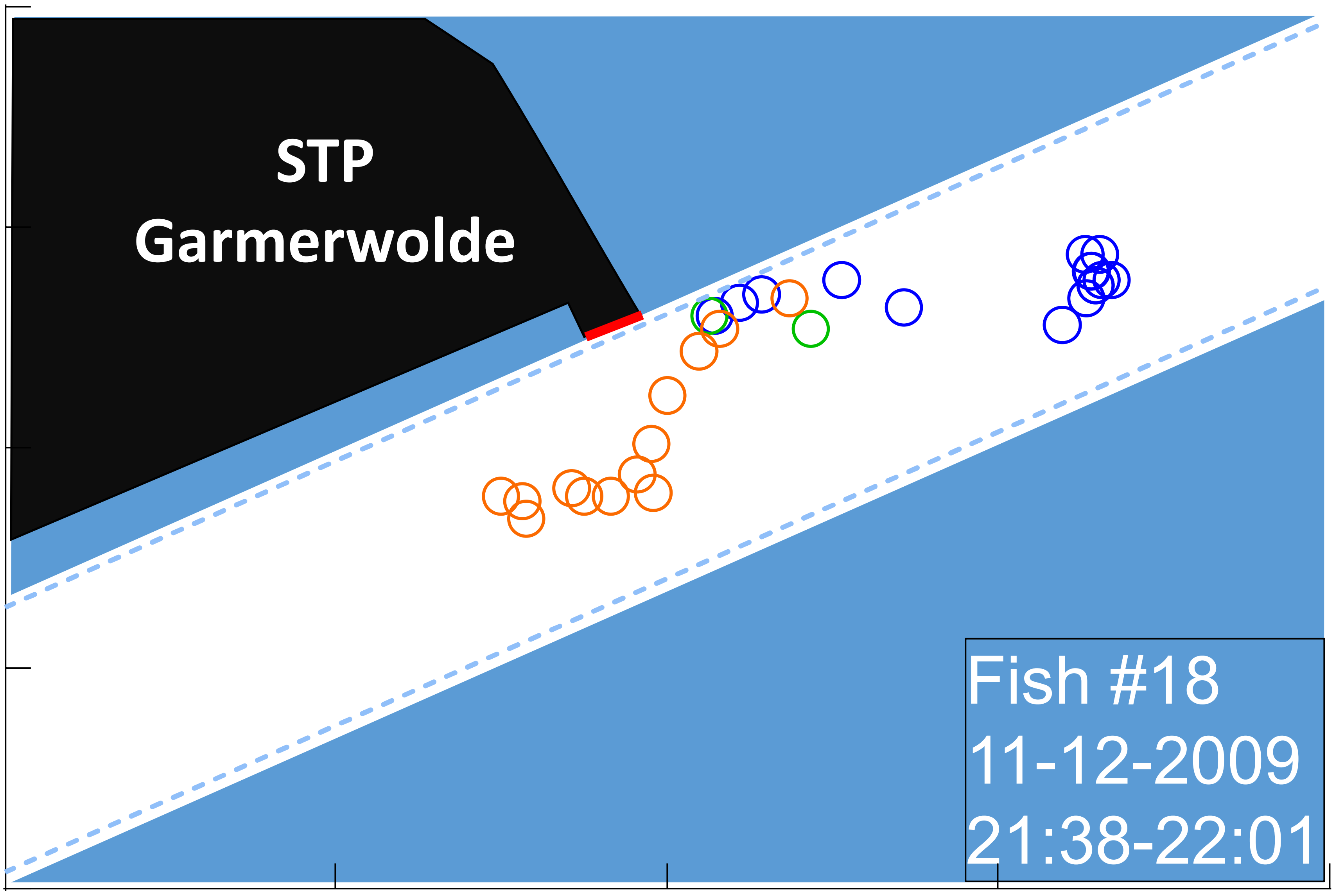
STP
Garmerwolde
Fish #18
11-12-2009
21:38-22:01

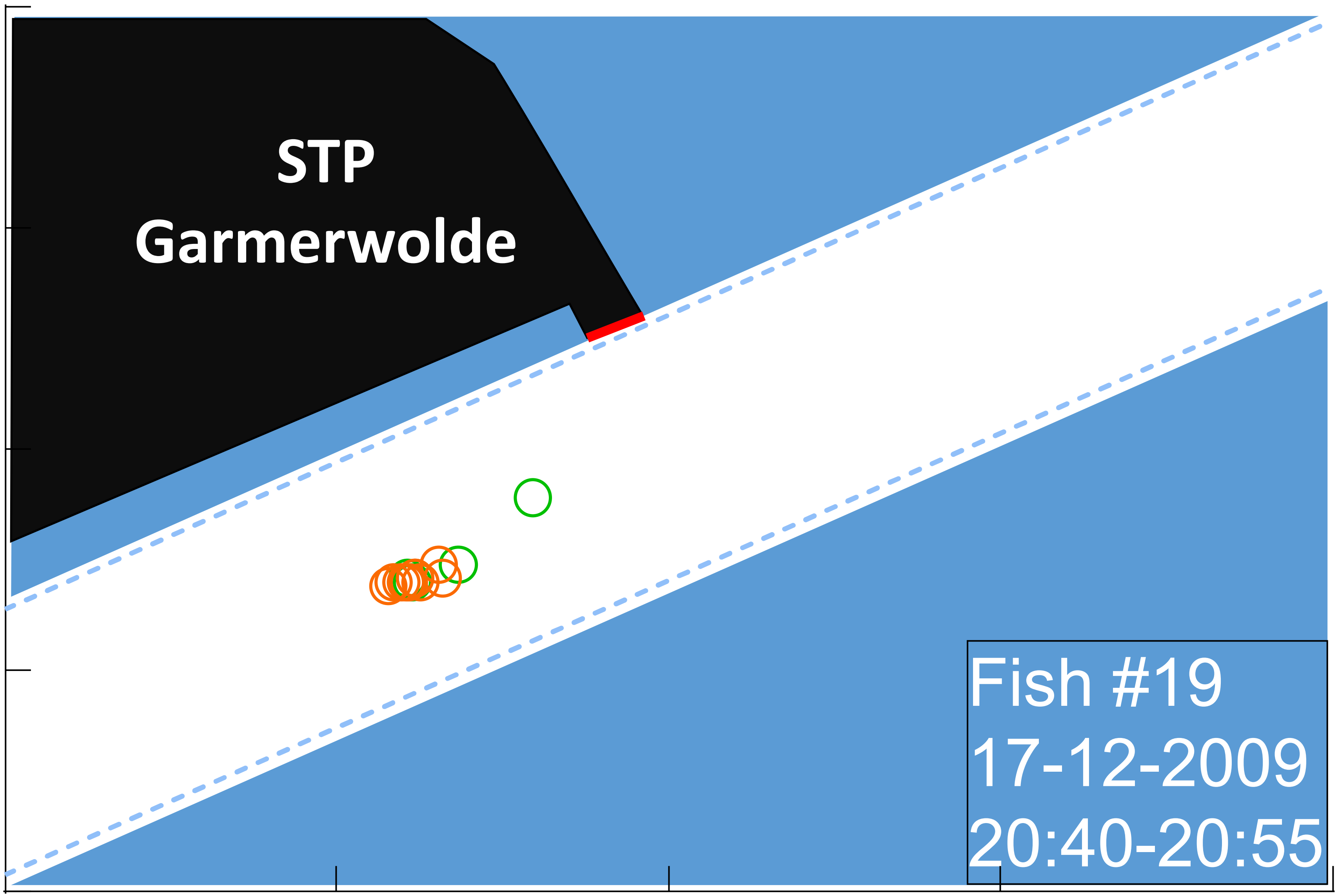
STP
Garmerwolde
Fish #19
17-12-2009
20:40-20:55

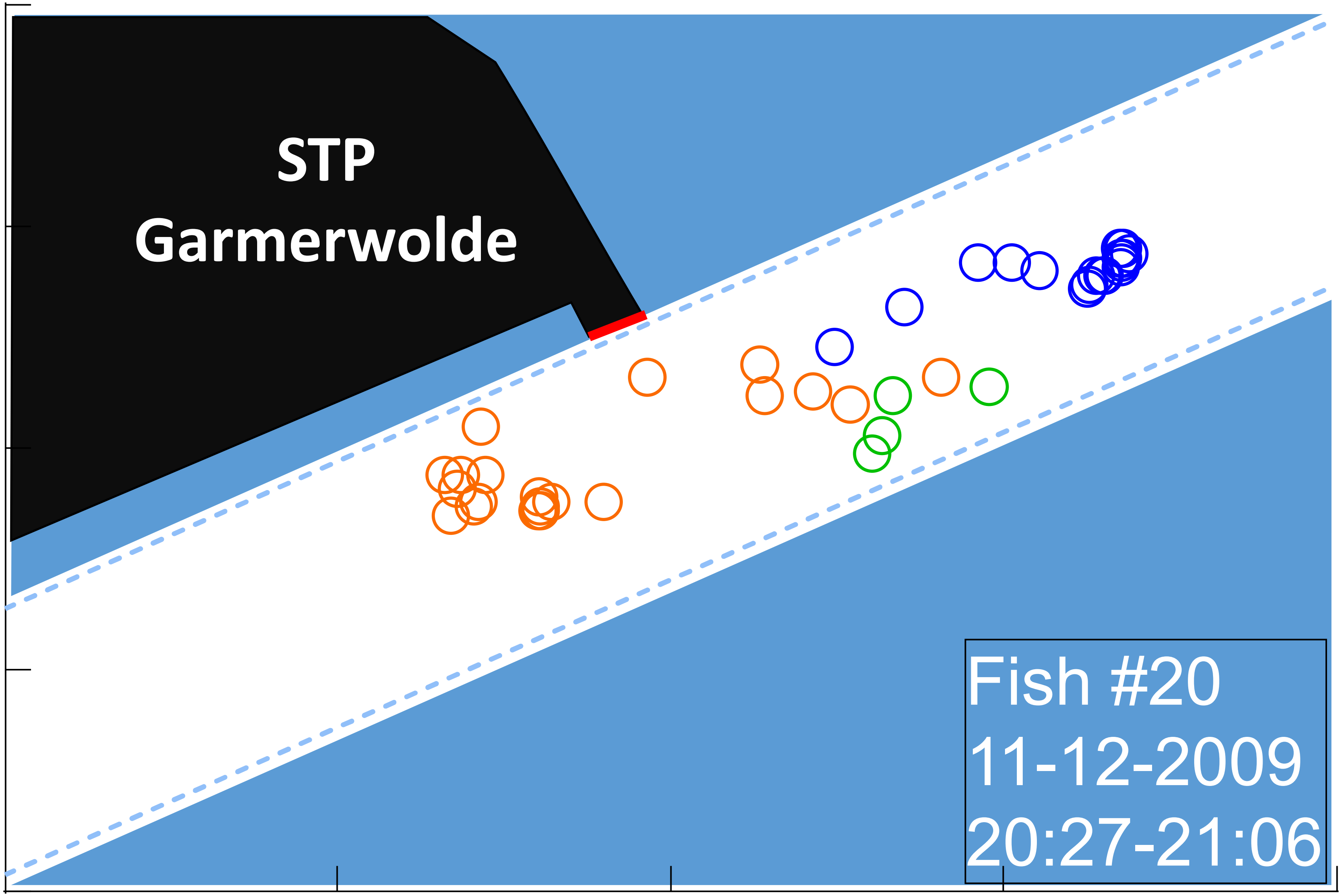

STP
Garmerwolde
Fish #20
11-12-2009
20:27-21:06
