# Supplementary material for: Wastewater plumes can act as non-physical barriers for migrating silver eel
Source: PLoS One. 2023 Jun 27;18(6):e0287189. doi: 10.1371/journal.pone.0287189 (PMC10298781; doi:10.1371/journal.pone.0287189)

Wastewater plumes can act as non-physical barriers for migrating eel.
A field study based on telemetry and plume modelling

H.V. Winter[1]*, O.A. van Keeken[1], F. Kleissen[2], E.M. Foekema[1,3]

[1]Wageningen Marine Research, IJmuiden, the Netherlands
[2]Deltares, Delft, the Netherlands
[3] Wageningen University, Marine Animal Ecology group, Wageningen, The Netherlands

*Corresponding author: erwin.winter@wur.nl

Supporting information
Eel movements 2010

Characterisation of behaviour 2010 silver eel

Eel 2010	Behavioural code	Short description
1	2D	Extensive movements outside plume
2	1B	No response, random turning
3	1C	No response, random movements
4	2C	Diversion at plume
5	2B	Turning, eventually passage
6	2D	Extensive movements outside plume
7	2A	Turning, no passage
8	2C	Diversion at plume
9	2C	Diversion at plume
10	1C	No response, random movements
11	1A	No response, passing through
12	1C	Extensive movements outside plume
13	2D	No response, random movements
14	1A	Diversion at plume
15	2C	Diversion at plume
16	2C	Diversion at plume
17	-	Not detected
18	2D	Extensive movements outside plume
19	1A	No response, passing through
20	1B	No response, random turning

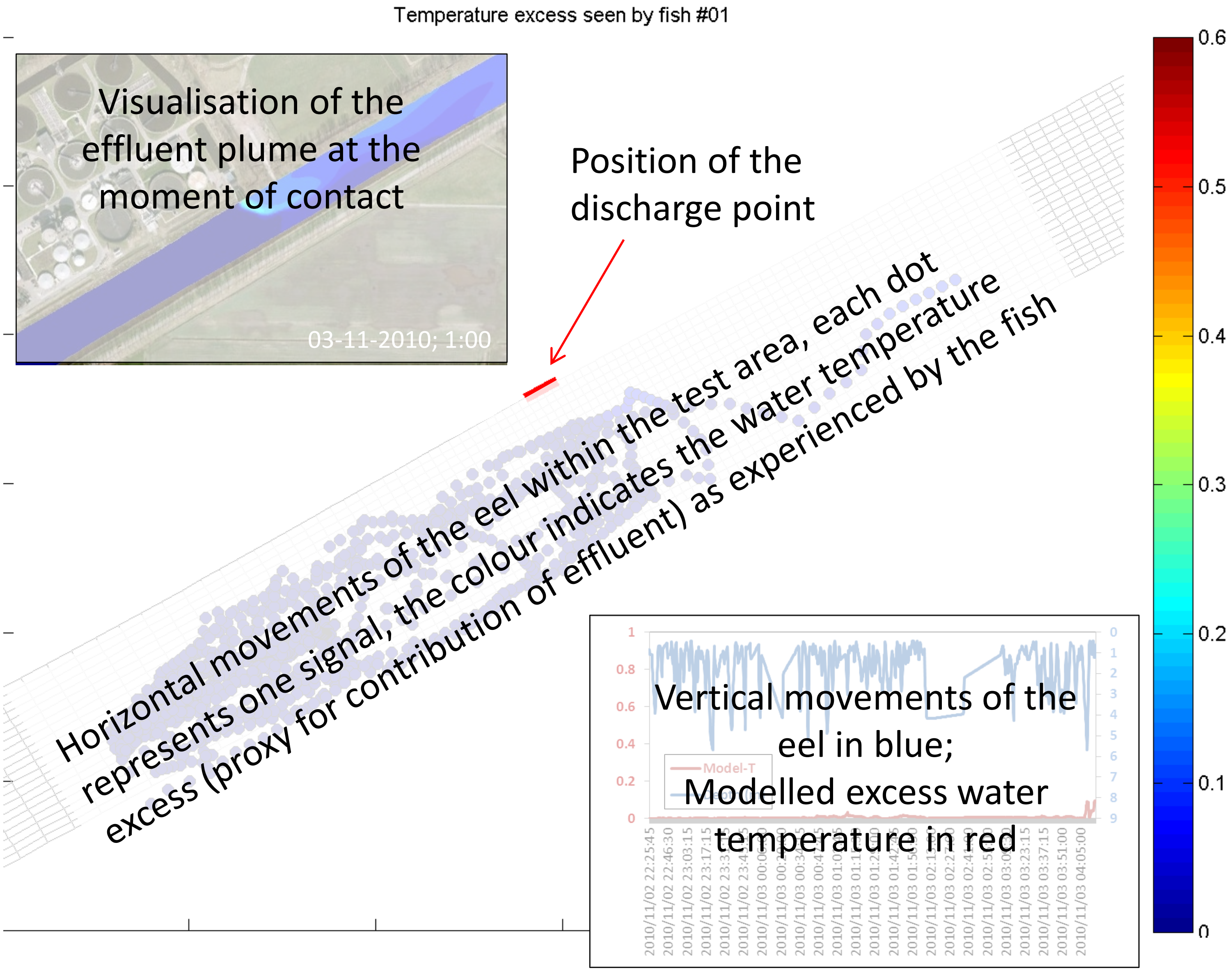

Temperature excess seen by fish #01
Visualisation of the effluent plume at the moment of contact
03-11-2010; 1:00
Position of the discharge point
Horizontal movements of the eel within the test area, each dot represents one signal, the colour indicates the water temperature excess (proxy for contribution of effluent) as experienced by the fish
Vertical movements of the eel in blue;
Modelled excess water temperature in red
Model-T
0.6
0.5
0.4
0.3
0.2
0.1
0

#01

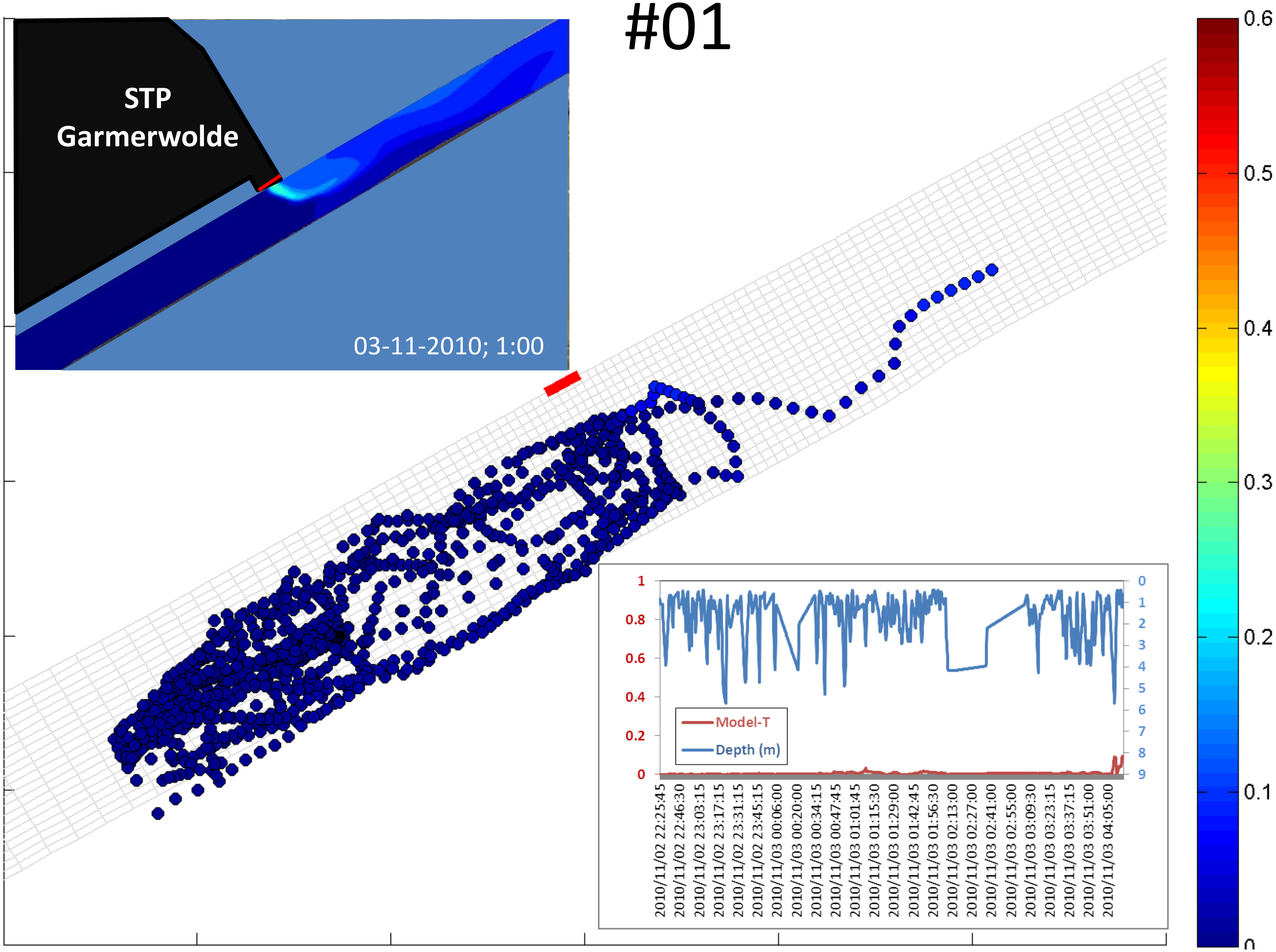

STP
Garmerwolde
03-11-2010; 1:00
Model-T
Depth (m)
0.6
0.5
0.4
0.3
0.2
0.1
0

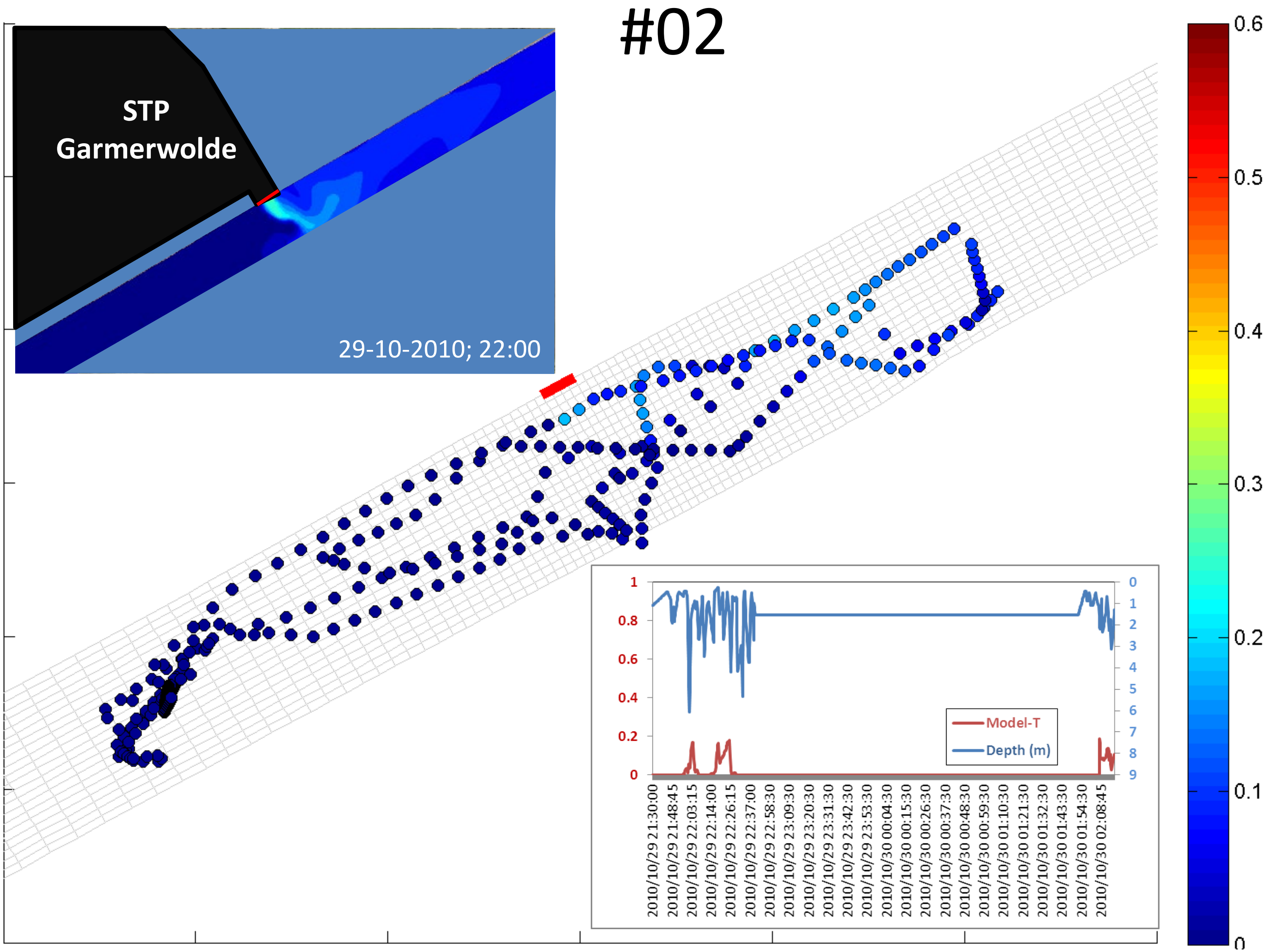
#02
STP
Garmerwolde
29-10-2010; 22:00
0.6
0.5
0.4
0.3
0.2
0.1
0
Model-T
Depth (m)
1
0.8
0.6
0.4
0.2
0
0
1
2
3
4
5
6
7
8
9
2010/10/29 21:30:00
2010/10/29 21:48:45
2010/10/29 22:03:15
2010/10/29 22:14:00
2010/10/29 22:26:15
2010/10/29 22:37:00
2010/10/29 22:58:30
2010/10/29 23:09:30
2010/10/29 23:20:30
2010/10/29 23:31:30
2010/10/29 23:42:30
2010/10/29 23:53:30
2010/10/30 00:04:30
2010/10/30 00:15:30
2010/10/30 00:26:30
2010/10/30 00:37:30
2010/10/30 00:48:30
2010/10/30 00:59:30
2010/10/30 01:10:30
2010/10/30 01:21:30
2010/10/30 01:32:30
2010/10/30 01:43:30
2010/10/30 01:54:30
2010/10/30 02:08:45

#03

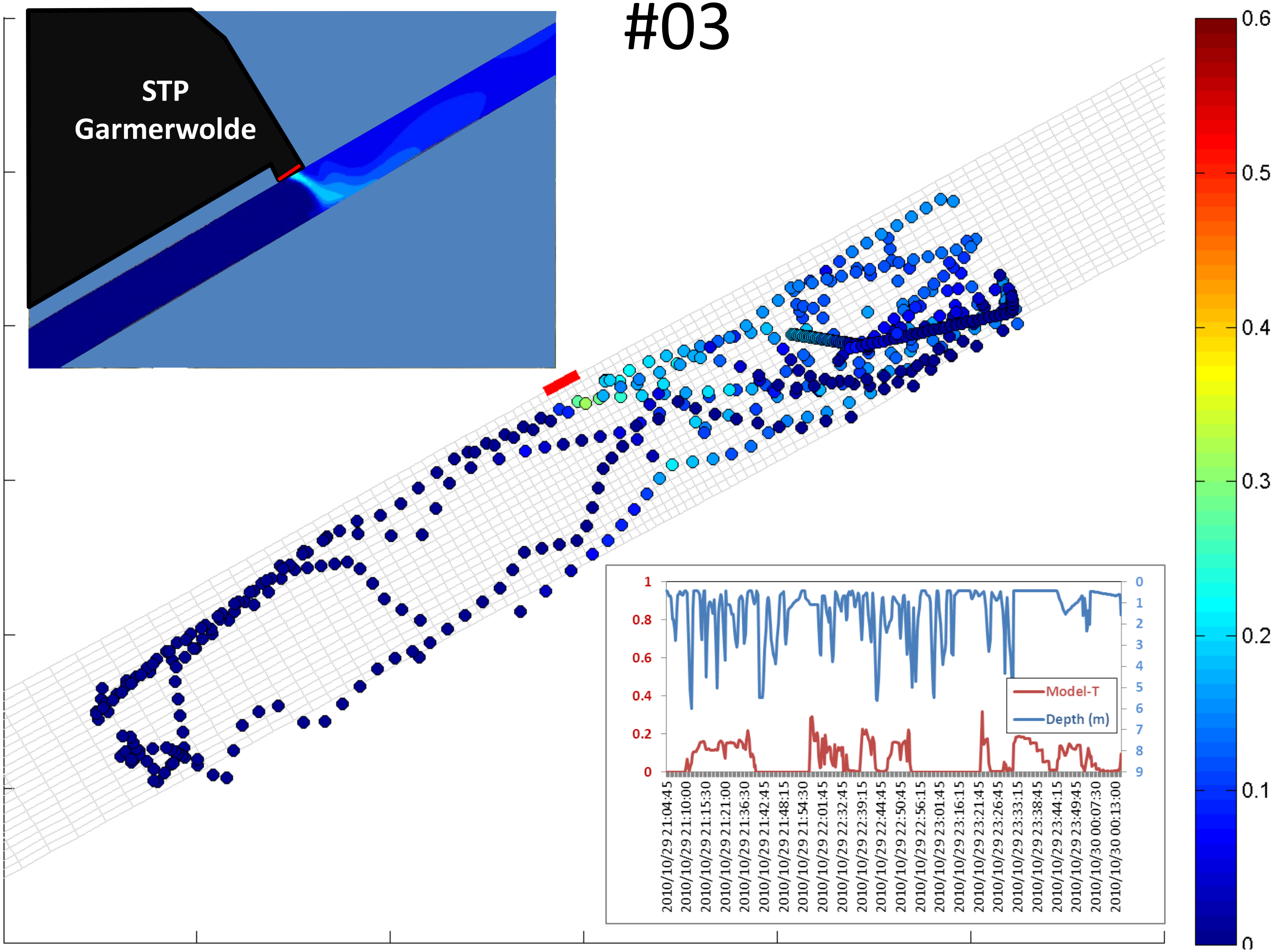

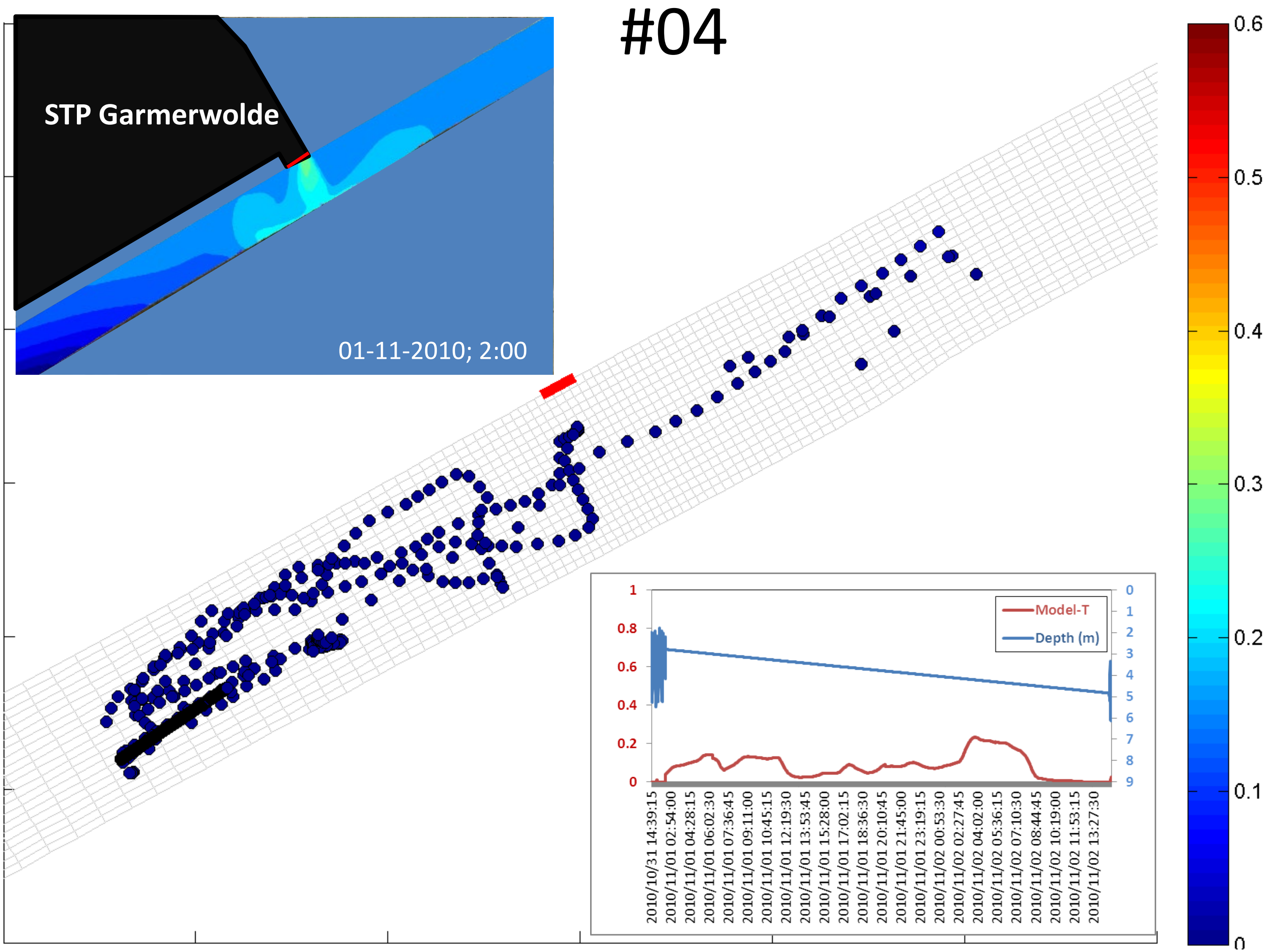

#04
STP Garmerwolde
01-11-2010; 2:00
Model-T
Depth (m)
0
0.1
0.2
0.3
0.4
0.5
0.6
0
0.2
0.4
0.6
0.8
1
0
1
2
3
4
5
6
7
8
9
2010/10/31 14:39:15
2010/11/01 02:54:00
2010/11/01 04:28:15
2010/11/01 06:02:30
2010/11/01 07:36:45
2010/11/01 09:11:00
2010/11/01 10:45:15
2010/11/01 12:19:30
2010/11/01 13:53:45
2010/11/01 15:28:00
2010/11/01 17:02:15
2010/11/01 18:36:30
2010/11/01 20:10:45
2010/11/01 21:45:00
2010/11/01 23:19:15
2010/11/02 00:53:30
2010/11/02 02:27:45
2010/11/02 04:02:00
2010/11/02 05:36:15
2010/11/02 07:10:30
2010/11/02 08:44:45
2010/11/02 10:19:00
2010/11/02 11:53:15
2010/11/02 13:27:30

#05

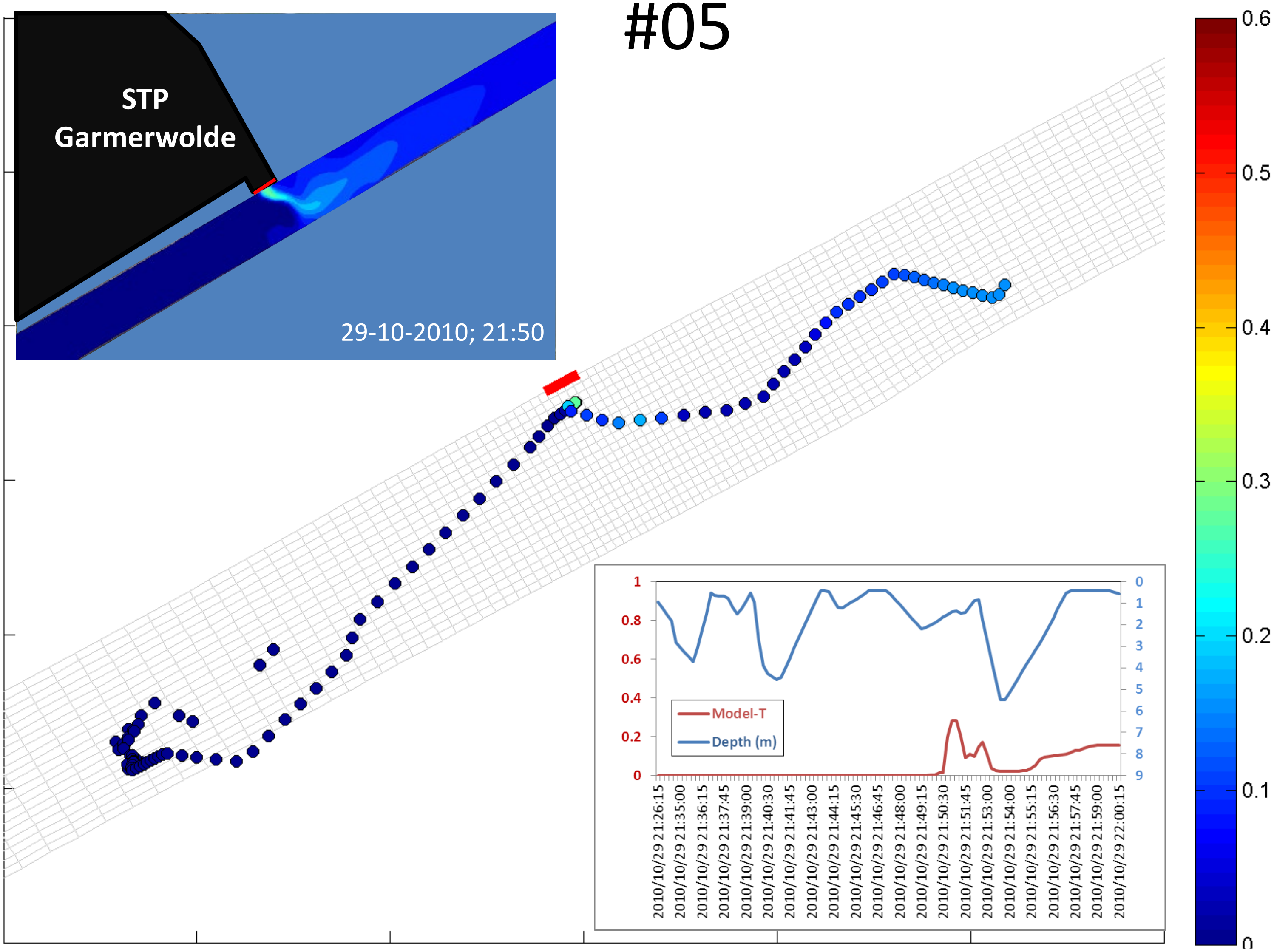
STP
Garmerwolde
29-10-2010; 21:50
Model-T
Depth (m)
0.6
0.5
0.4
0.3
0.2
0.1
0

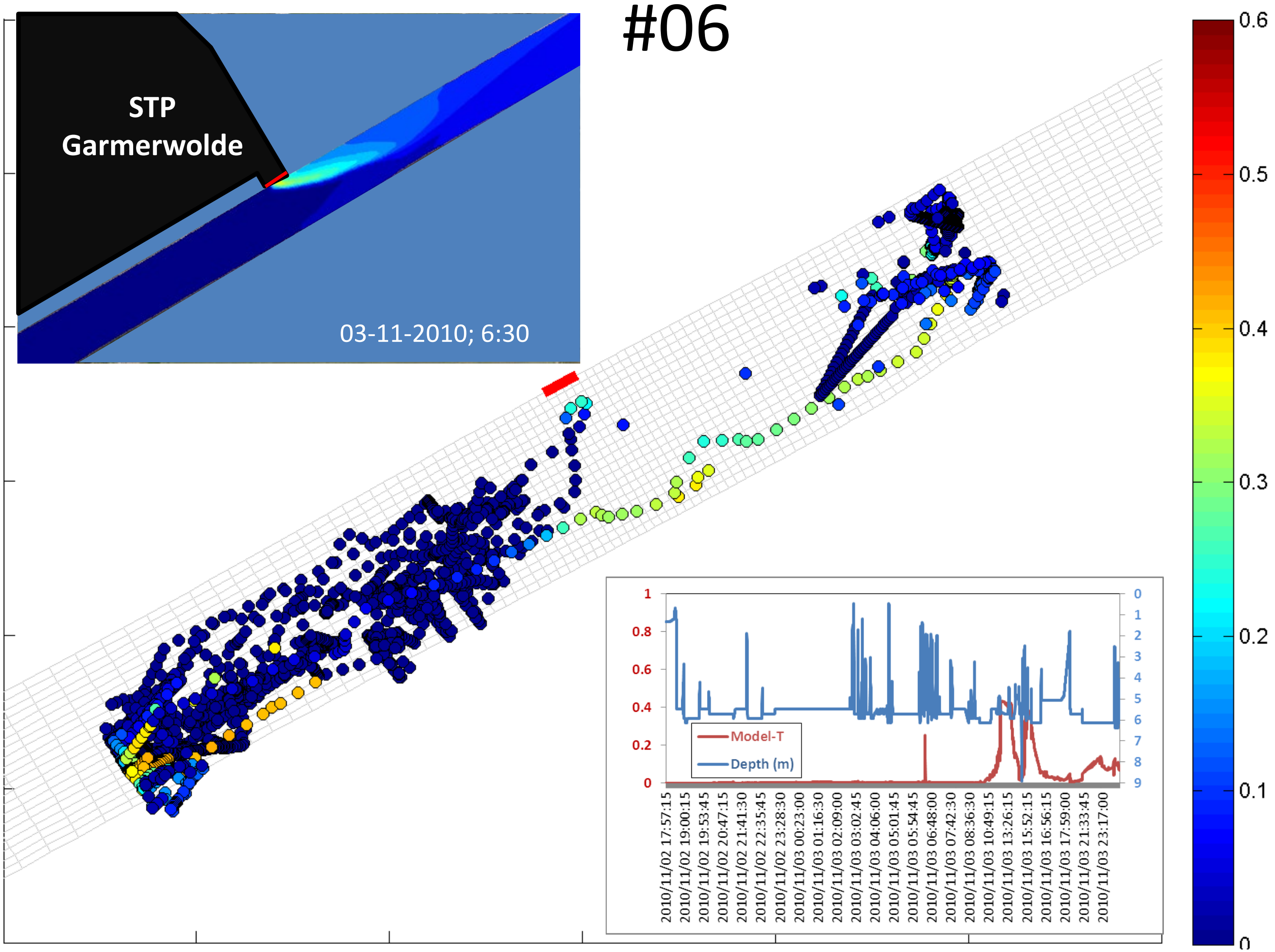
#06
STP
Garmerwolde
03-11-2010; 6:30
Model-T
Depth (m)
2010/11/02 17:57:15
2010/11/02 19:00:15
2010/11/02 19:53:45
2010/11/02 20:47:15
2010/11/02 21:41:30
2010/11/02 22:35:45
2010/11/02 23:28:30
2010/11/03 00:23:00
2010/11/03 01:16:30
2010/11/03 02:09:00
2010/11/03 03:02:45
2010/11/03 04:06:00
2010/11/03 05:01:45
2010/11/03 05:54:45
2010/11/03 06:48:00
2010/11/03 07:42:30
2010/11/03 08:36:30
2010/11/03 10:49:15
2010/11/03 13:26:15
2010/11/03 15:52:15
2010/11/03 16:56:15
2010/11/03 17:59:00
2010/11/03 21:33:45
2010/11/03 23:17:00

#07

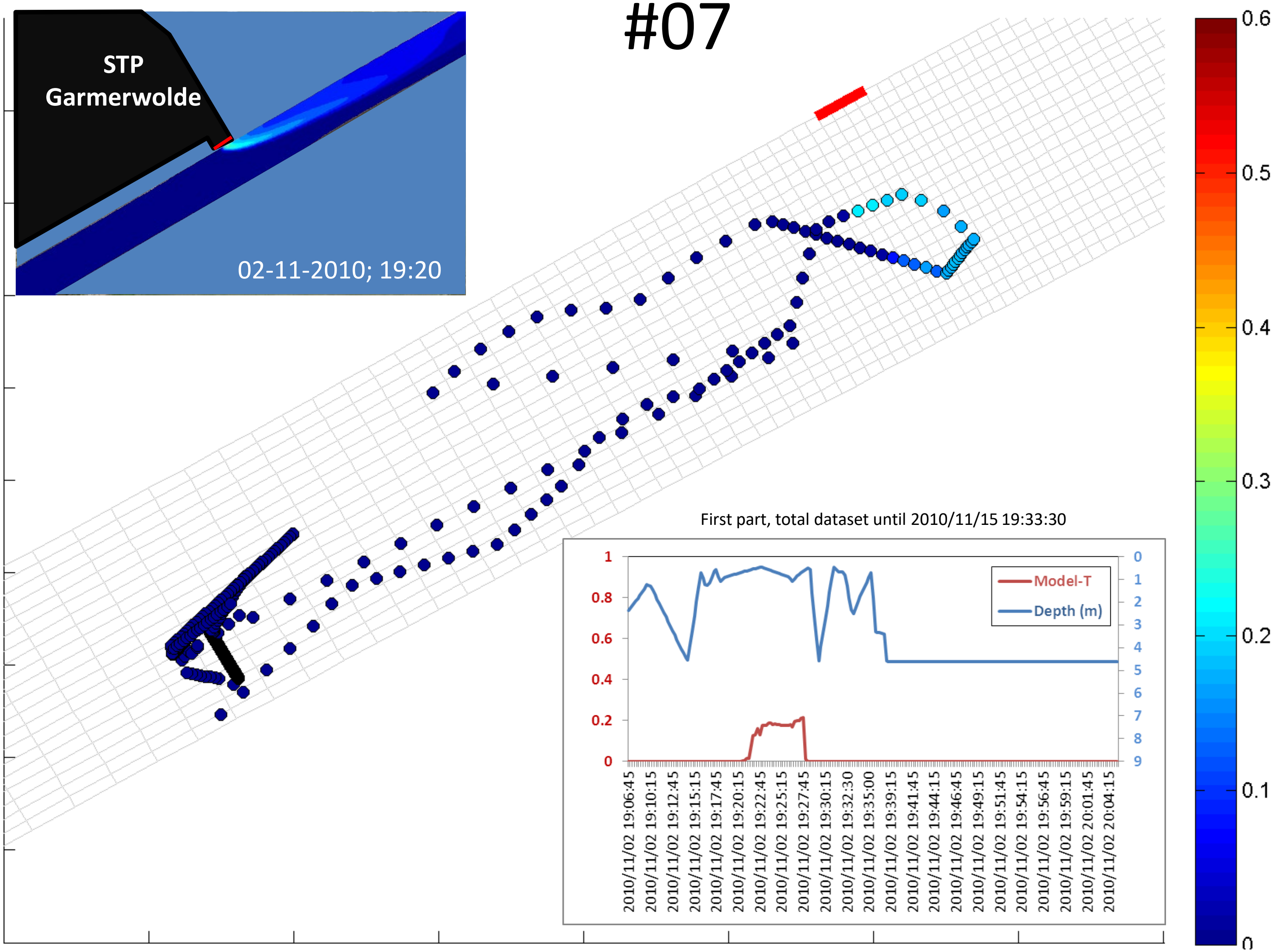
STP
Garmerwolde
02-11-2010; 19:20
First part, total dataset until 2010/11/15 19:33:30
Model-T
Depth (m)
0.6
0.5
0.4
0.3
0.2
0.1
0

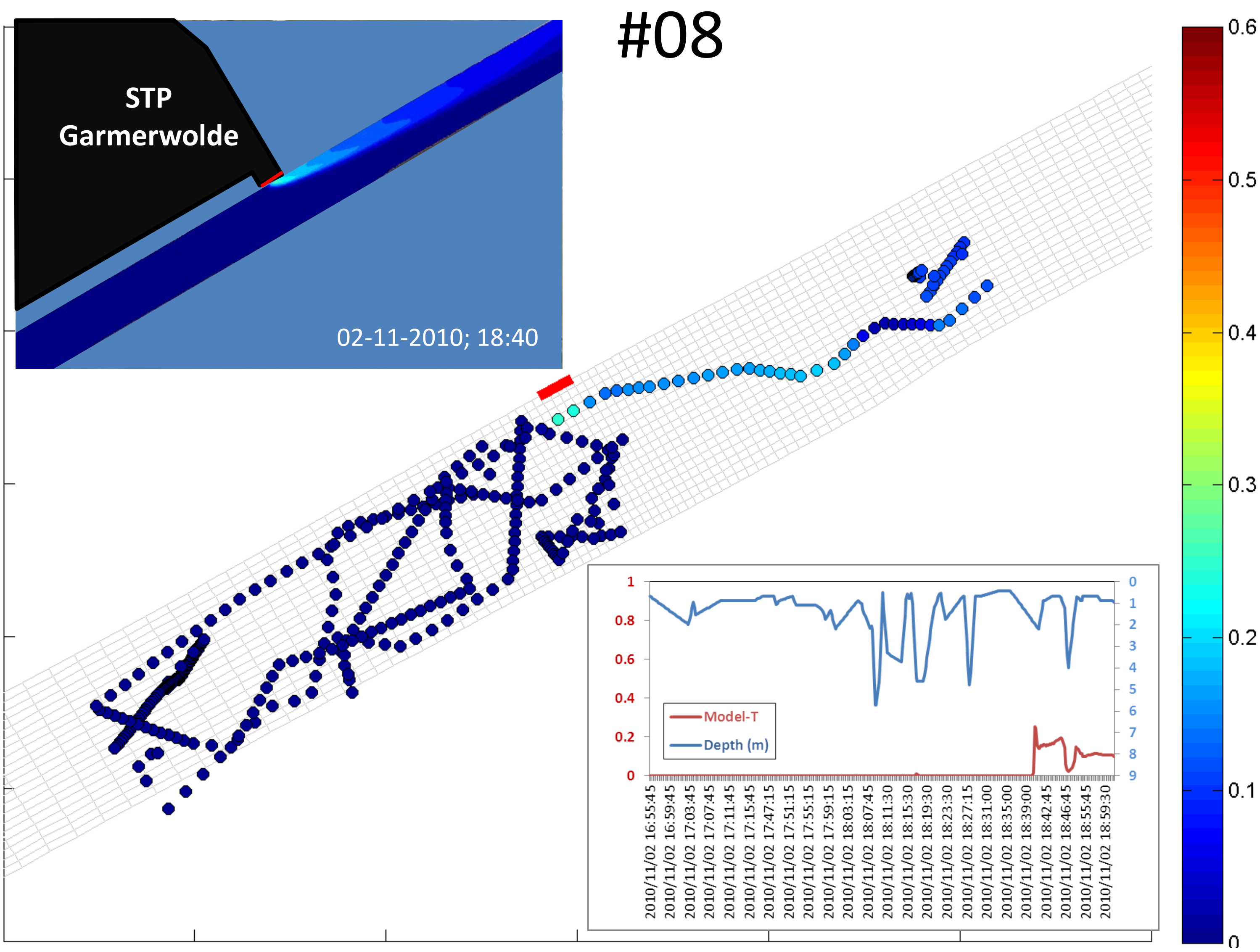

#08
STP
Garmerwolde
02-11-2010; 18:40
0.6
0.5
0.4
0.3
0.2
0.1
0
Model-T
Depth (m)
1
0.8
0.6
0.4
0.2
0
0
1
2
3
4
5
6
7
8
9
2010/11/02 16:55:45
2010/11/02 16:59:45
2010/11/02 17:03:45
2010/11/02 17:07:45
2010/11/02 17:11:45
2010/11/02 17:15:45
2010/11/02 17:47:15
2010/11/02 17:51:15
2010/11/02 17:55:15
2010/11/02 17:59:15
2010/11/02 18:03:15
2010/11/02 18:07:45
2010/11/02 18:11:30
2010/11/02 18:15:30
2010/11/02 18:19:30
2010/11/02 18:23:30
2010/11/02 18:27:15
2010/11/02 18:31:00
2010/11/02 18:35:00
2010/11/02 18:39:00
2010/11/02 18:42:45
2010/11/02 18:46:45
2010/11/02 18:55:45
2010/11/02 18:59:30

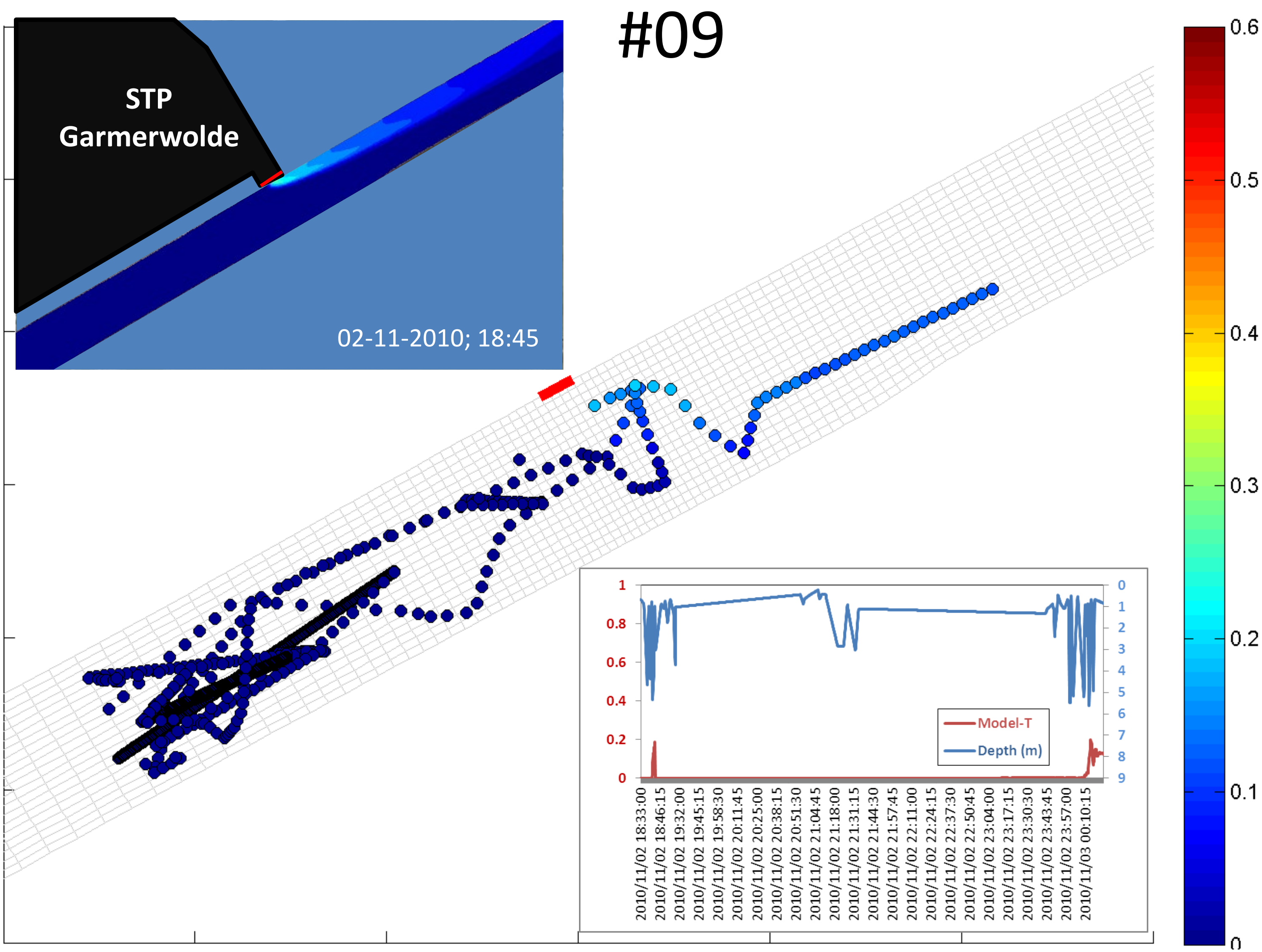
#09
STP
Garmerwolde
02-11-2010; 18:45
Model-T
Depth (m)
0
0.1
0.2
0.3
0.4
0.5
0.6

#10

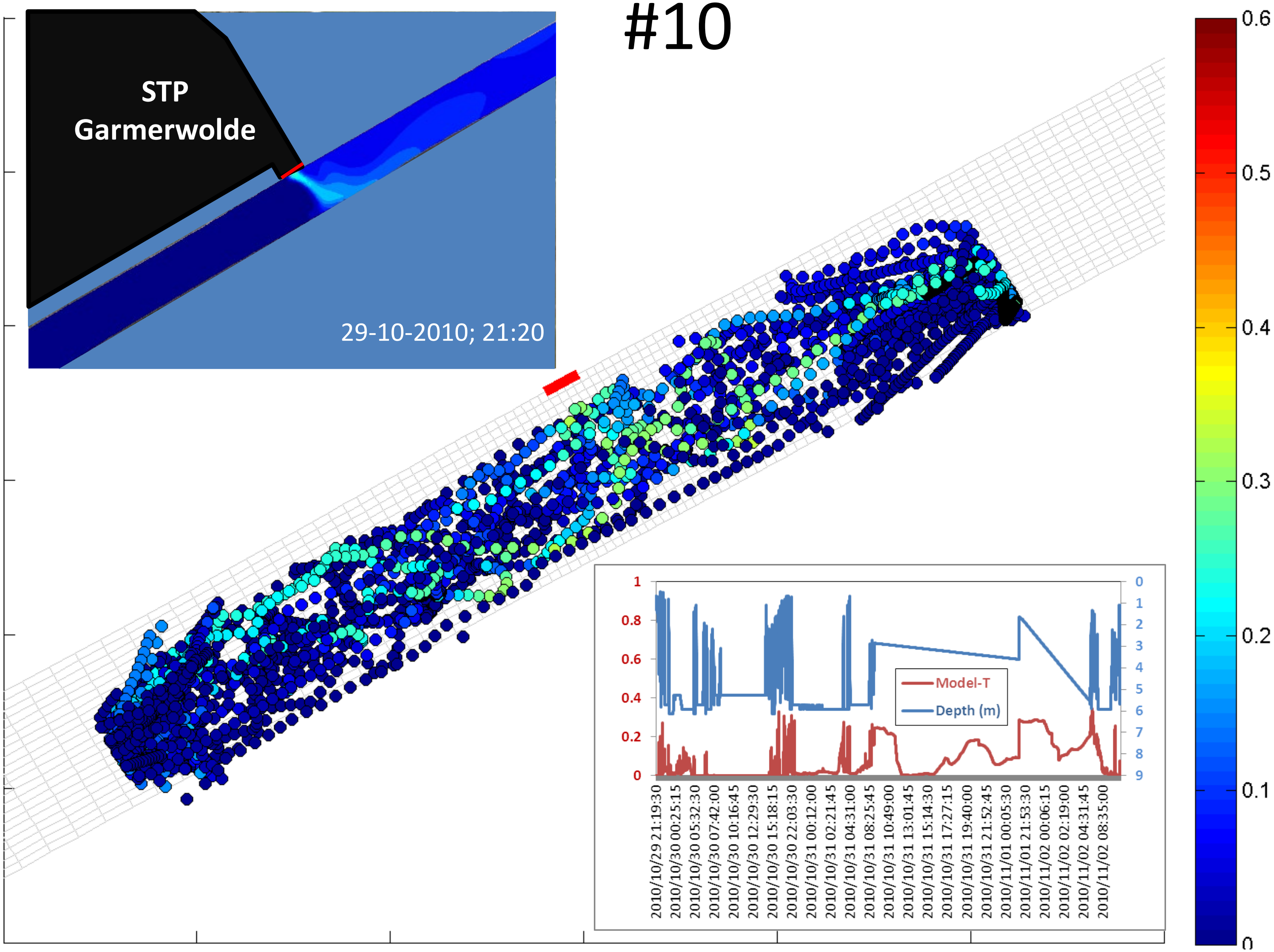
STP
Garmerwolde
29-10-2010; 21:20
Model-T
Depth (m)
0.6
0.5
0.4
0.3
0.2
0.1
0

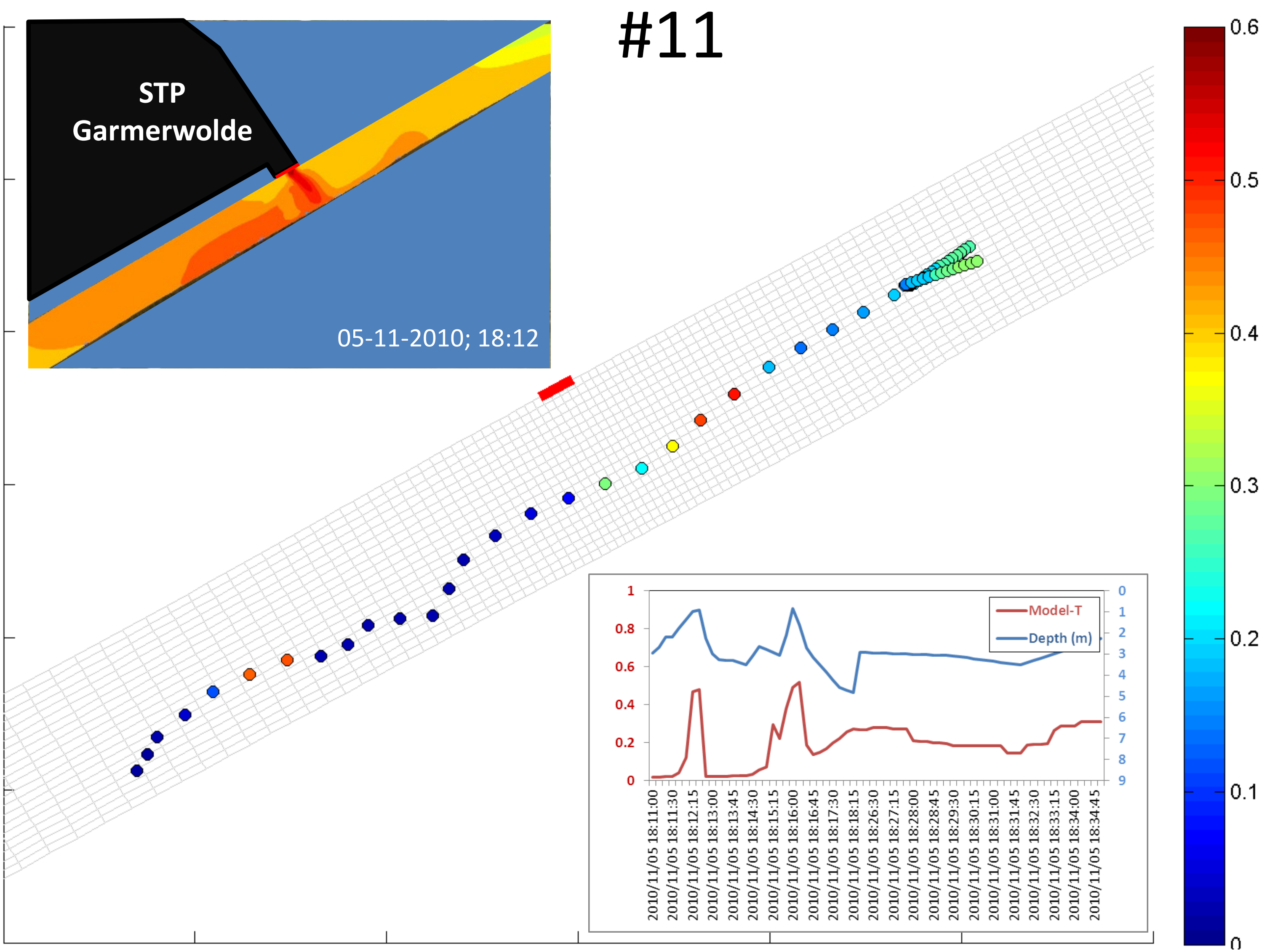
#11
STP
Garmerwolde
05-11-2010; 18:12
Model-T
Depth (m)
1
0.8
0.6
0.4
0.2
0
0
1
2
3
4
5
6
7
8
9
2010/11/05 18:11:00
2010/11/05 18:11:30
2010/11/05 18:12:15
2010/11/05 18:13:00
2010/11/05 18:13:45
2010/11/05 18:14:30
2010/11/05 18:15:15
2010/11/05 18:16:00
2010/11/05 18:16:45
2010/11/05 18:17:30
2010/11/05 18:18:15
2010/11/05 18:26:30
2010/11/05 18:27:15
2010/11/05 18:28:00
2010/11/05 18:28:45
2010/11/05 18:29:30
2010/11/05 18:30:15
2010/11/05 18:31:00
2010/11/05 18:31:45
2010/11/05 18:32:30
2010/11/05 18:33:15
2010/11/05 18:34:00
2010/11/05 18:34:45
0.6
0.5
0.4
0.3
0.2
0.1
0

#12

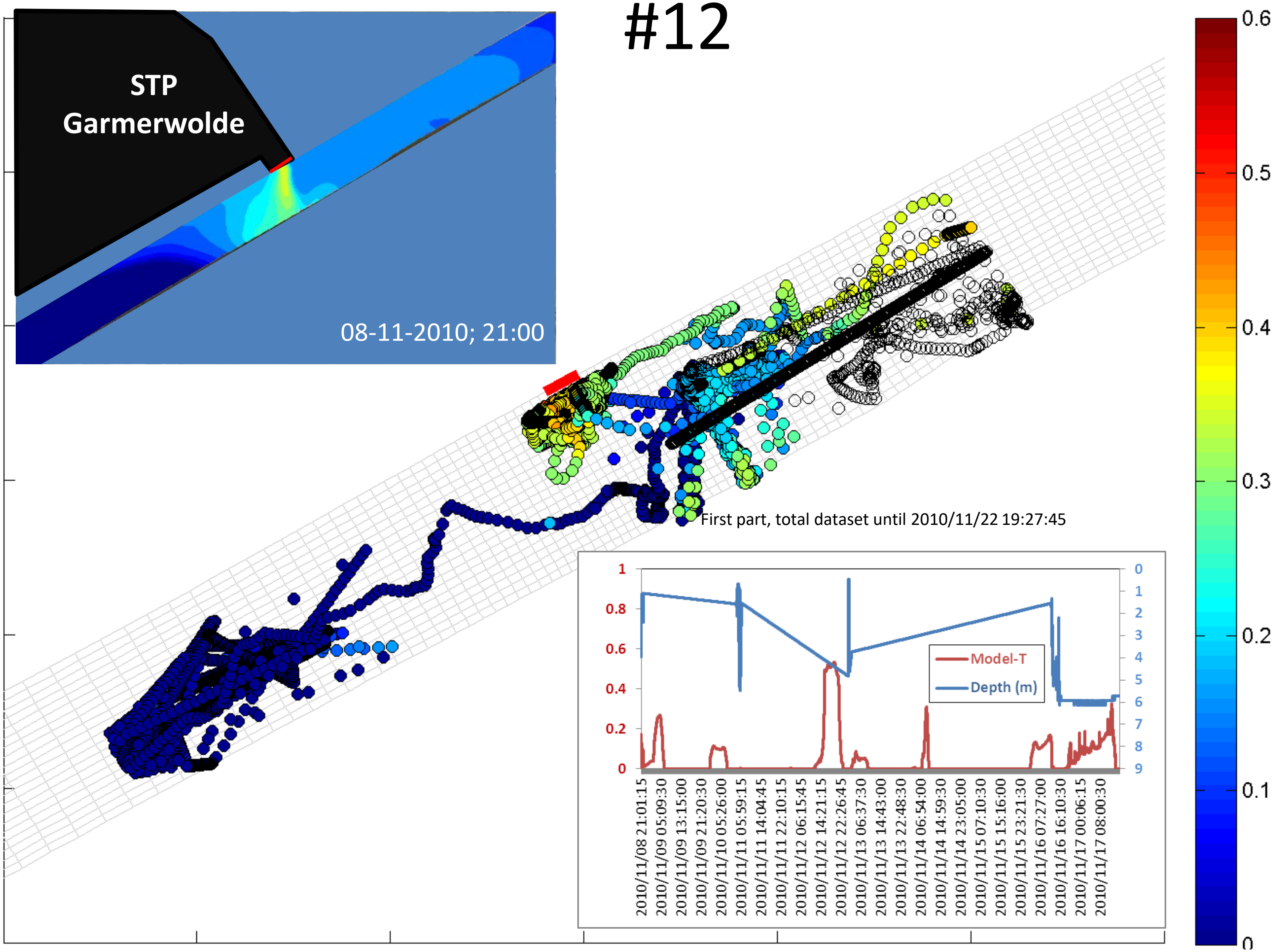
STP
Garmerwolde
08-11-2010; 21:00
First part, total dataset until 2010/11/22 19:27:45
Model-T
Depth (m)
1
0.8
0.6
0.4
0.2
0
0
1
2
3
4
5
6
7
8
9
2010/11/08 21:01:15
2010/11/09 05:09:30
2010/11/09 13:15:00
2010/11/09 21:20:30
2010/11/10 05:26:00
2010/11/11 05:59:15
2010/11/11 14:04:45
2010/11/11 22:10:15
2010/11/12 06:15:45
2010/11/12 14:21:15
2010/11/12 22:26:45
2010/11/13 06:37:30
2010/11/13 14:43:00
2010/11/13 22:48:30
2010/11/14 06:54:00
2010/11/14 14:59:30
2010/11/14 23:05:00
2010/11/15 07:10:30
2010/11/15 15:16:00
2010/11/15 23:21:30
2010/11/16 07:27:00
2010/11/16 16:10:30
2010/11/17 00:06:15
2010/11/17 08:00:30
0.6
0.5
0.4
0.3
0.2
0.1
0

#13

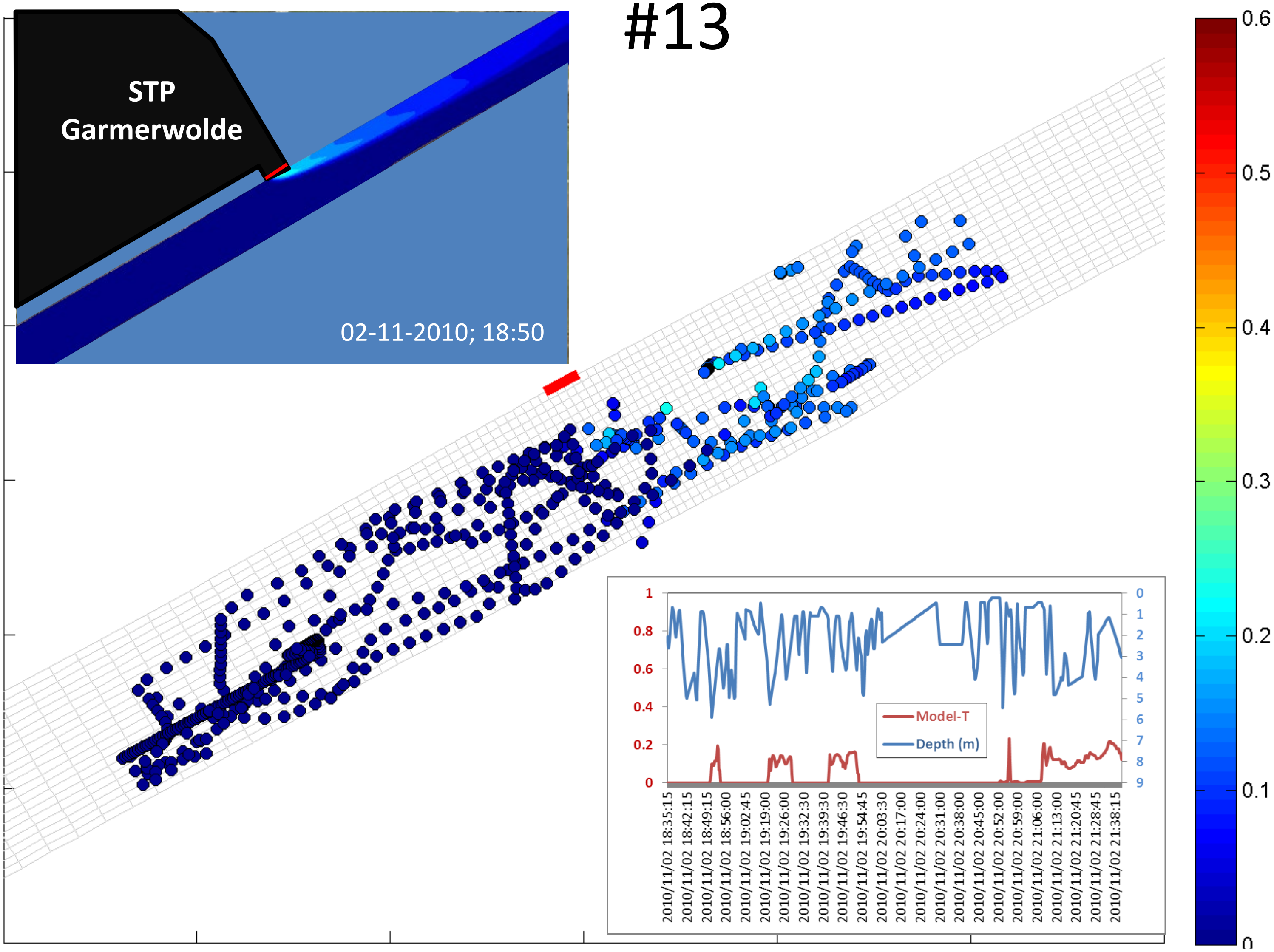

STP
Garmerwolde
02-11-2010; 18:50
Model-T
Depth (m)
0.6
0.5
0.4
0.3
0.2
0.1
0

#14

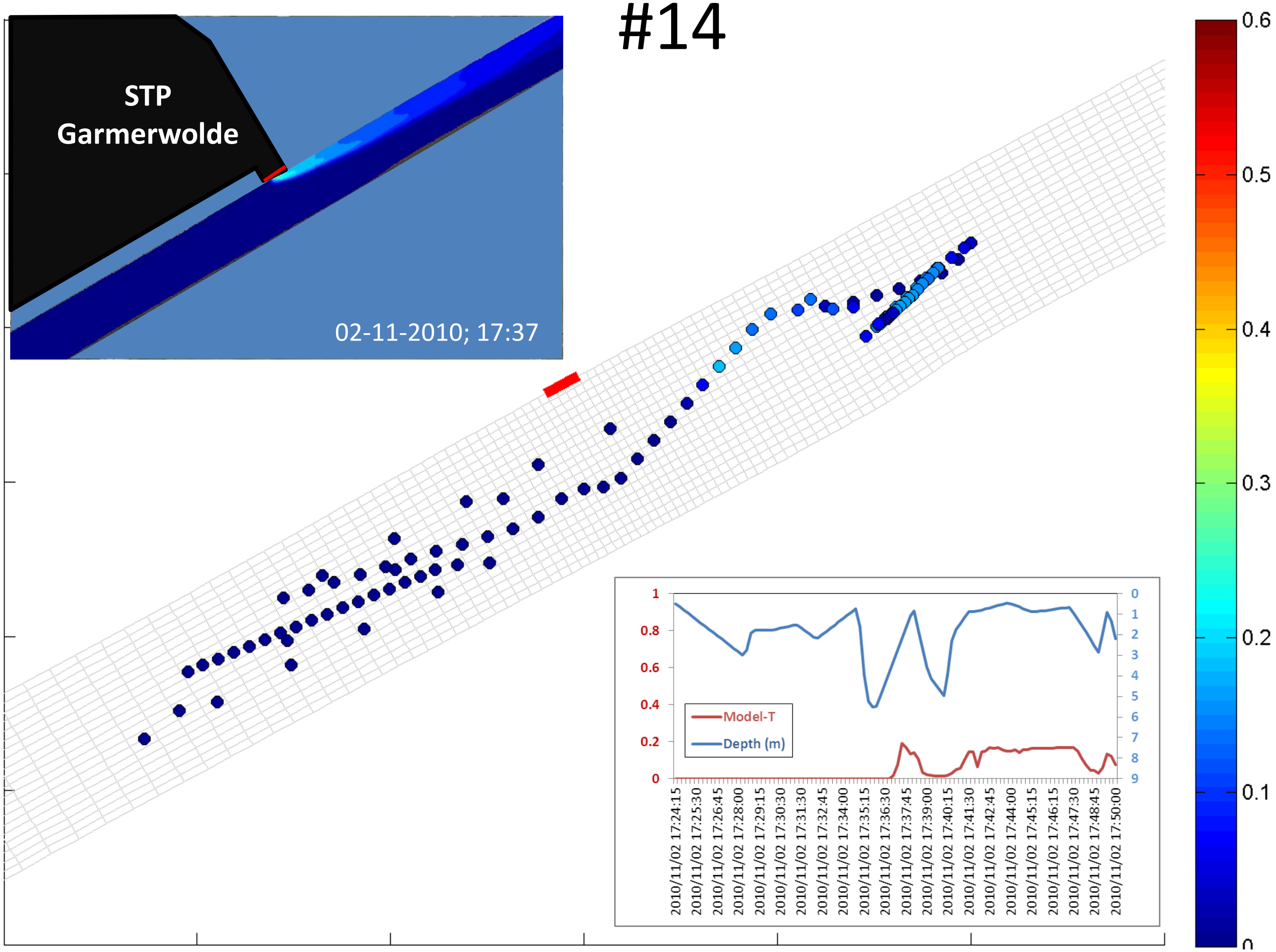
STP
Garmerwolde
02-11-2010; 17:37
Model-T
Depth (m)
0.6
0.5
0.4
0.3
0.2
0.1
0

#15

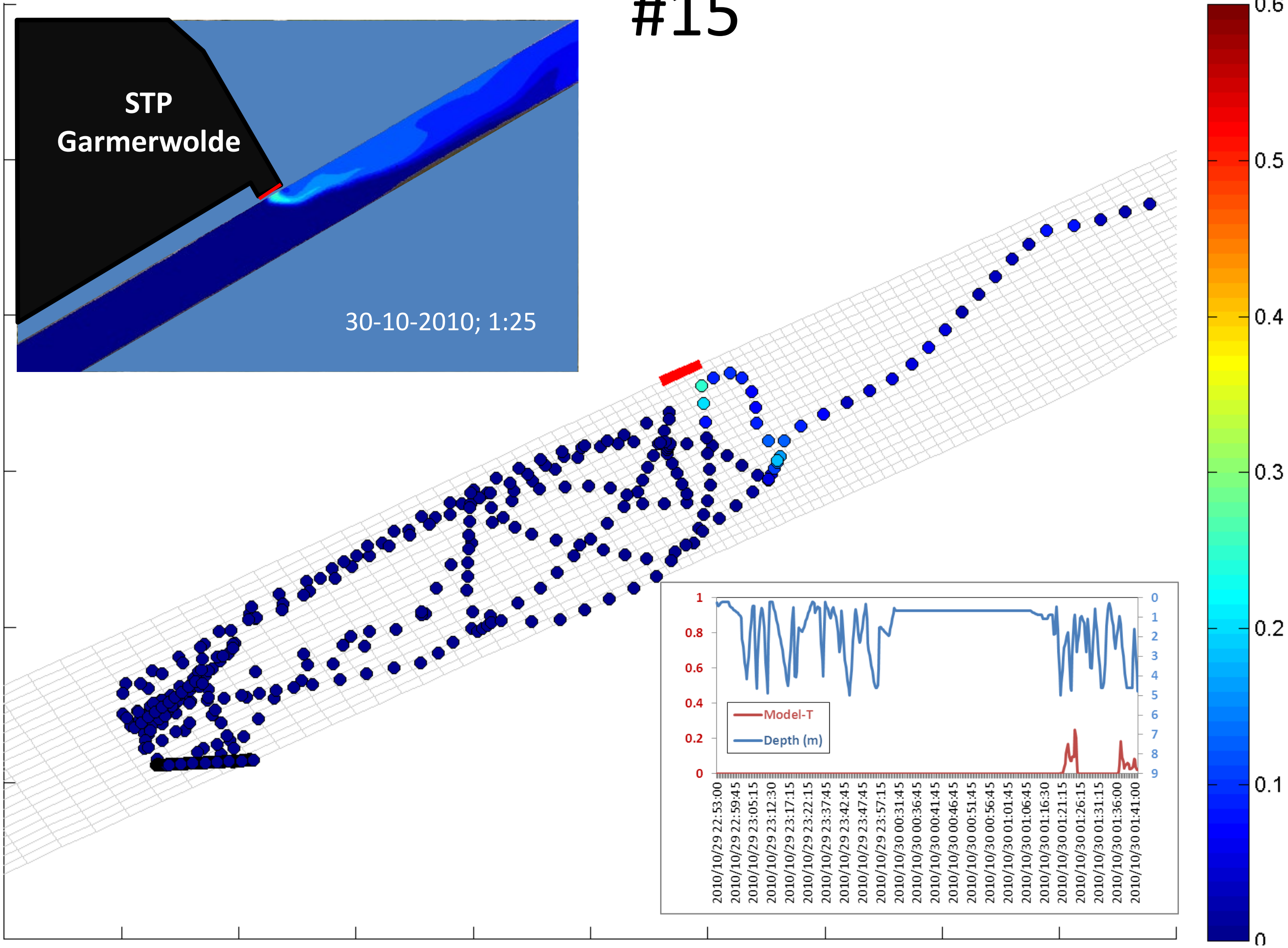
STP
Garmerwolde
30-10-2010; 1:25
Model-T
Depth (m)
0.6
0.5
0.4
0.3
0.2
0.1
0

#16

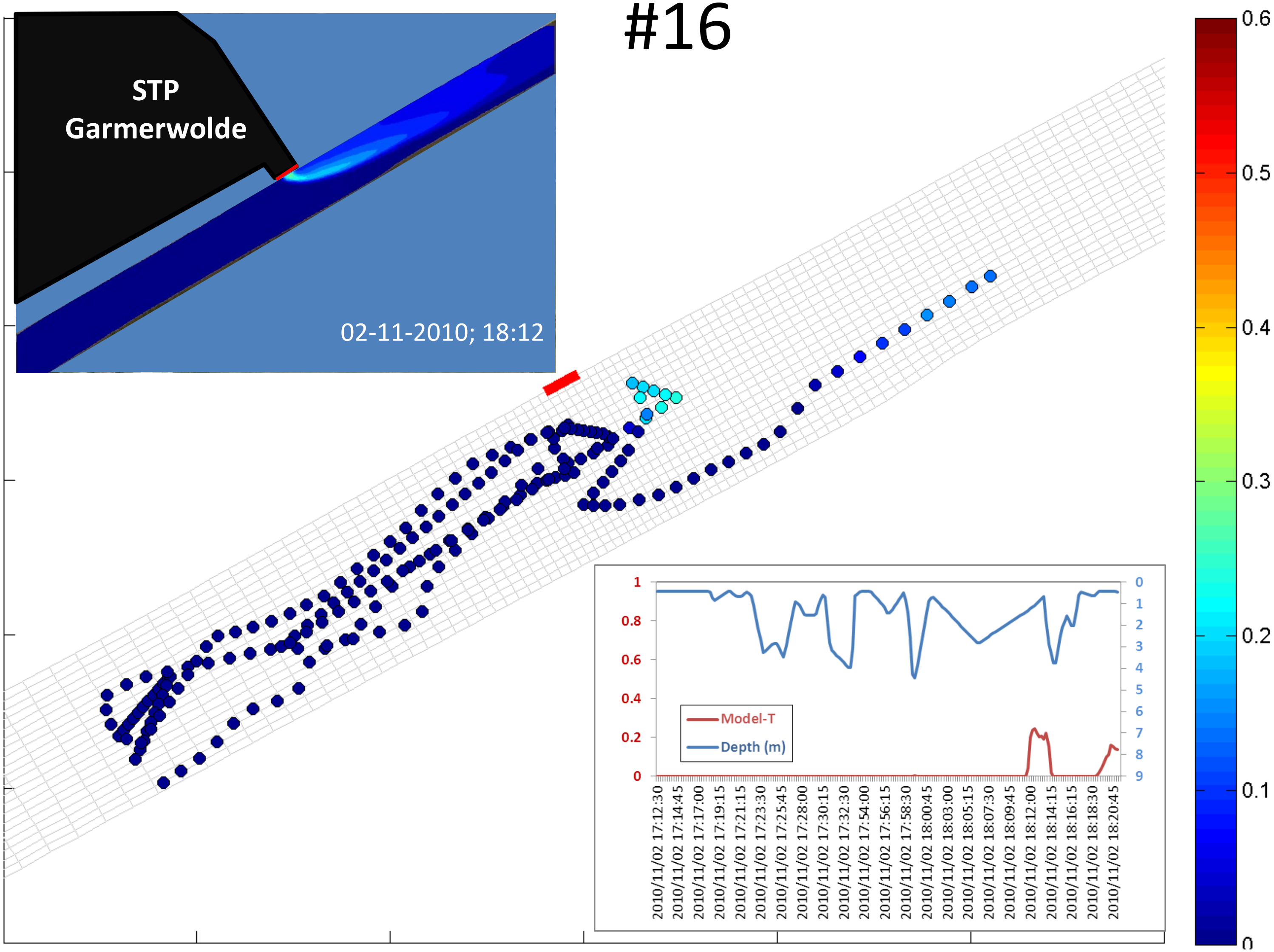

STP
Garmerwolde
02-11-2010; 18:12
Model-T
Depth (m)
0.6
0.5
0.4
0.3
0.2
0.1
0

#17

No data

#18

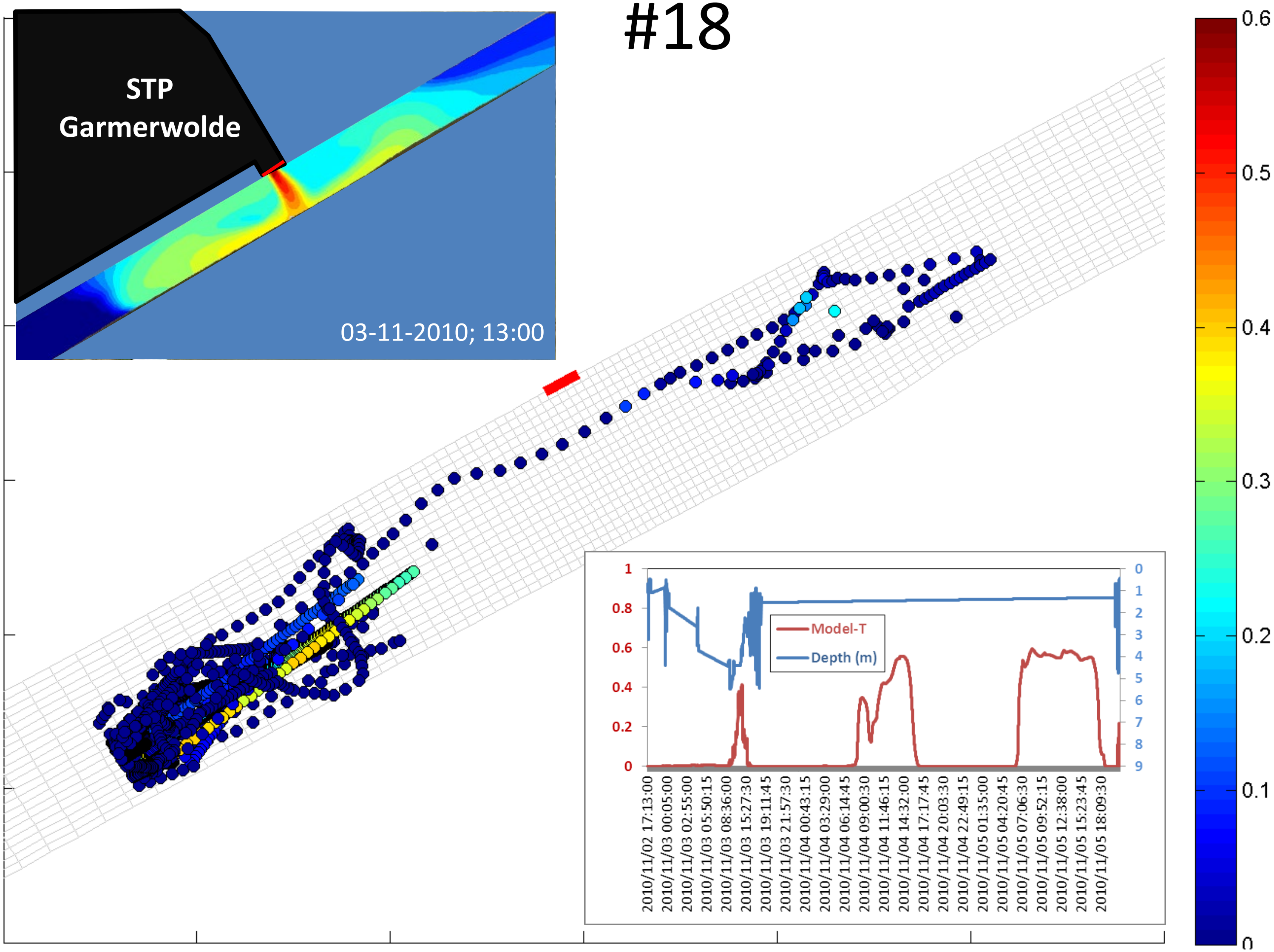
STP
Garmerwolde
03-11-2010; 13:00
Model-T
Depth (m)
0.6
0.5
0.4
0.3
0.2
0.1
0

#19

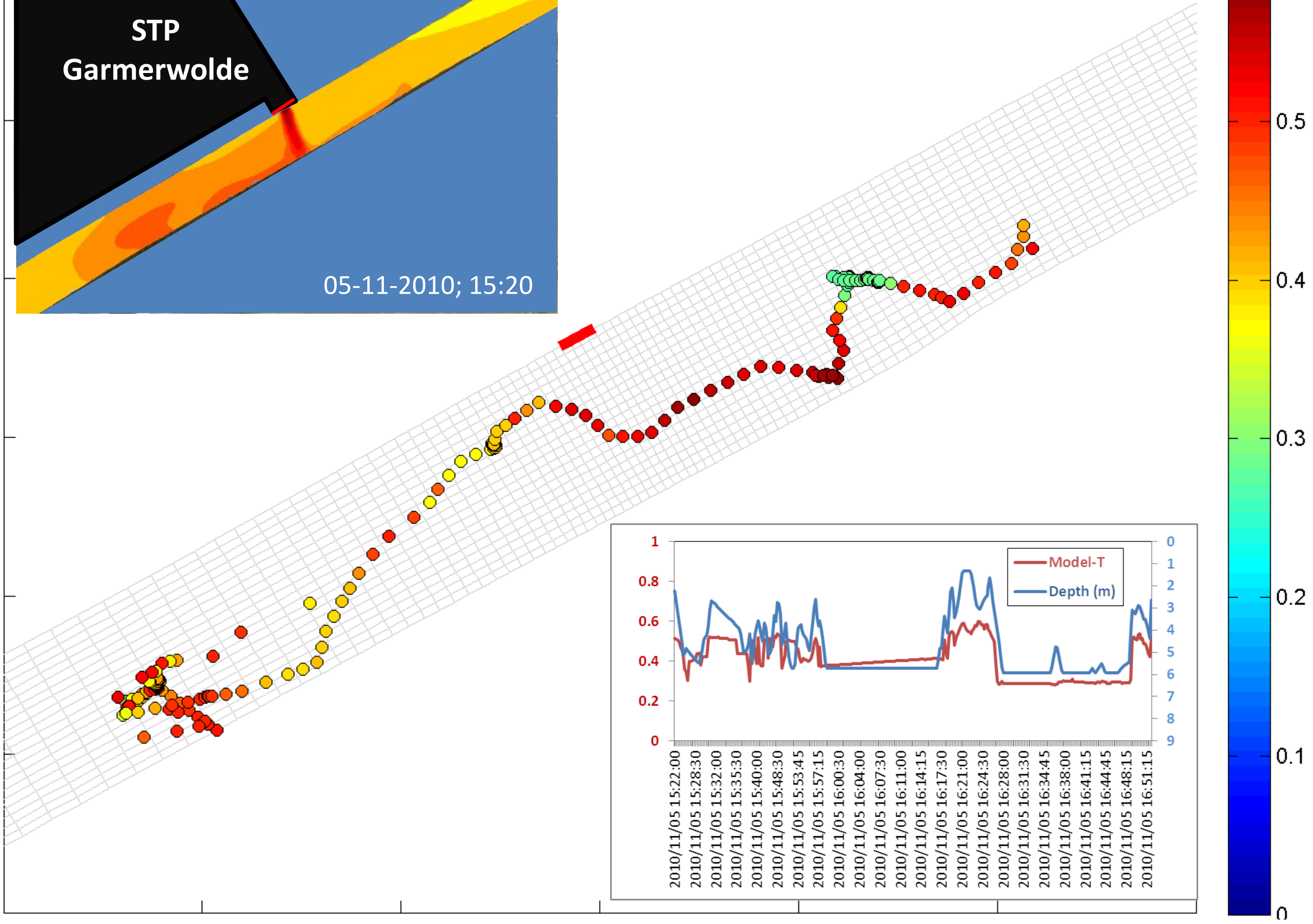
STP
Garmerwolde
05-11-2010; 15:20
Model-T
Depth (m)
1
0.8
0.6
0.4
0.2
0
0
1
2
3
4
5
6
7
8
9
2010/11/05 15:22:00
2010/11/05 15:28:30
2010/11/05 15:32:00
2010/11/05 15:35:30
2010/11/05 15:40:00
2010/11/05 15:48:30
2010/11/05 15:53:45
2010/11/05 15:57:15
2010/11/05 16:00:30
2010/11/05 16:04:00
2010/11/05 16:07:30
2010/11/05 16:11:00
2010/11/05 16:14:15
2010/11/05 16:17:30
2010/11/05 16:21:00
2010/11/05 16:24:30
2010/11/05 16:28:00
2010/11/05 16:31:30
2010/11/05 16:34:45
2010/11/05 16:38:00
2010/11/05 16:41:15
2010/11/05 16:44:45
2010/11/05 16:48:15
2010/11/05 16:51:15
0.6
0.5
0.4
0.3
0.2
0.1
0

#20

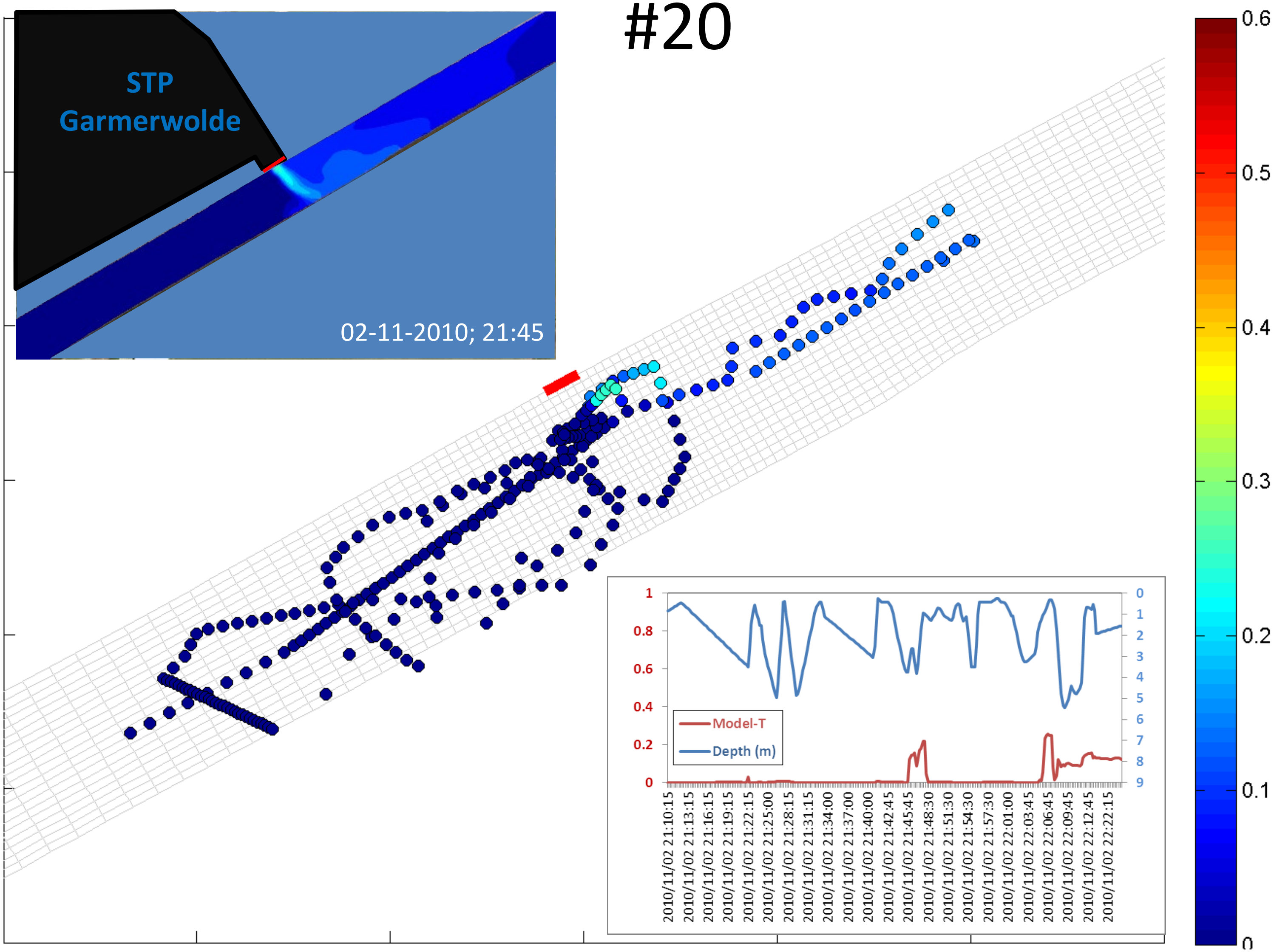

Supplement: S2 Appendix — (PDF) [file pone.0287189.s002.pdf]
