# Supplementary material for: Wastewater plumes can act as non-physical barriers for migrating silver eel
Source: PLoS One. 2023 Jun 27;18(6):e0287189. doi: 10.1371/journal.pone.0287189 (PMC10298781; doi:10.1371/journal.pone.0287189)
Supplement: Attachment — Submitted filename: A1 Reviewers reply Rebuttal resubmission.docx [file pone.0287189.s003.docx]

Dear editor,

We hereby submit a revised version of our manuscript ‘Wastewater Plumes Can Act as Non-physical Barriers for Migrating Eel. A Field Study Based on Telemetry and Plume Modelling‘ for publication in PlosOne.

Thank you for sharing the reviews of both reviewers. We carefully considered them and improved the manuscript to the best of our ability. However, we find the comments of both reviewers very general without clearly pointing out what needs to be addressed specifically. In addition, at some points we disagree with the provided reviews, which we explain in our rebuttal to each reviewer below.

The comments from two other reviewers that we received on our earlier submission of this manuscript to PLOSONE (‘PONE-D-16-50845’, that was accepted with minor revisions as we were informed by email by Caroline Durif on 18/05/2017), were much more detailed, concrete and constructive and helped us to improve the manuscript. For completeness, we also included these reviews and our rebuttals in our submission of our revised manuscript.

We would ask you to take these previous reviews into consideration with your decision as well.

We hope you will consider our manuscript for publication in PlosOne, and look forward to your response.

Kind Regards, on behalf of all co-authors,

Erwin Winter (Dr. Hendrik V. Winter)

**Reviewer #1: General Comment:**
The study investigated the behavioral responses of 40 acoustically-tagged silver eel
(Anguilla) were monitored in situ, when confronting a sewage treatment plant effluent plume
during their downstream migration in the canal Eems (The Netherlands).
Specific Comments

1. The manuscript needs significant improvement in terms of language. A proofreading is required.

*Authors: this seems a general comment but no specific lines or problems were mentioned by the reviewer. We’ve had it proofread at our earlier revised version.*

2. The research lacks novelty. The author needs to explain and emphasize more the innovation of
this work in the introduction.

*Authors: We conducted an extensive literature survey and there is hardly any paper on the behavioural response of free ranging migrating fish to non-physical plumes that can act as barriers. As mentioned in the introduction, only one other paper on this topic (on salmon not on eel), has been published. Therefore we strongly disagree that our paper lacks novelty.*

3. Starting of abstract seems not smooth, need major revision of abstract by including
brief methodology, results, and findings of the manuscript.

*Authors: Abstract was revised accordingly.*

4. Introduction section was written briefly, please add more literature about the research
methodology. Furthermore, a comparison should be made over the operating conditions and yields in the literature rather than general information.

*Authors: to our opinion our introduction is not briefly. Also there are no more studies on this specific topic, so extra literature could not be added on methodology. The telemetry methodology we used is standard scientific state-of the art, which was therefore referenced in the M&M and not in the Introduction.*

The original aspect of the study should be emphasized. In addition, the aim of the study should be stated in more detail.

*Authors: to our opinion the aim of the study is clearly stated in the introduction. So for us it is unclear what the reviewers means with ‘more detail’.*

5. Please provide more details map for the study area by using satellite map. Replace for Fig.1.

*Authors: In response to the reviewers of the previous version of our manuscript we showed a map with the information that is needed to understand our set-up, setting and situation of the field study. To our opinion more detail will only distract for the purpose of this figure, and is not needed to understand our paper. The reviewer is not explaining why more details are needed.*

6. The methodology was not based on reliable references.

*Authors: This comment is unclear to us, we used telemetry techniques to measure the behaviour of free ranging eel in the field, which was referenced, and modelling to reconstruct plume traits in time, which was referenced as well.*

7. The authors should compare the results with those of the previous researches, and this
comparison should be presented in an individual table.

*Authors: as mentioned no previous studies where done on eel. There are no similar or related studies that allow a quantified comparison of our results.*

8. The references need to be checked to make sure they are complete.

*Authors: we have done this, so we do not know why the reviewer makes this comment. If the reviewer finds that there are errors in the references, he/she should indicate which are wrong or lacking.*

**Reviewer #2:**

The abstract was written in such style, at some positions it seems that, it giving suggestions instead of giving results .....

abstract should be in comprehensive style that express the result in the attractive and concise way.

Abstract is lengthy and should be rewrite in well mannered.

*Authors: abstract was revised. However results were given, no suggestions.*

why e.g. used in the square bracket along citation number? if there are examples then first somewhat mention then give references.

*Authors: we do not understand this comment.*

which types of chemicals are normally there that eel fishes confronted?

*Authors: The scope of our study was to determine if effluent plumes could hamper or affect downstream migration of silver eel and if these plumes could act as non-physical barriers. These effluents contain a mixture of numerous chemical compounds as we point out in our discussion. To determine to which specific compounds these eels respond to, requires extensive laboratory studies that could be the focus of follow-up studies, but is far beyond the feasibility of what could be done in this study.*

if attraction is there in plume for eels then it means it favours the migration?

*Authors: we do not understand this comment.*

Laboratory studies indicated that fish could detect a broad range of compounds in the water column, here what does meant of laboratory studies and what is mean of the this sentence 'fish could detect a broad range of compounds in the water column'

*Authors: we do not understand this comment.*

For what this study is useful in future?

*Authors: We think we addressed this with our statements at the end of Discussion and Conclusions, and in the summary: ‘When discharge points cannot be avoided, reduced or restricted to areas that are not preferred as safe migration routes, discharge points can be designed such, that the chance is limited that a waterway is (temporarily) impacted over its full width.’*

In figure 3, Oct instead of Okt

*Authors: Indeed, we changed it.*
